# Mechanism of O_2_/NO-Promoted Oxidative C–C Bond Cleavage in Linear Alkanes

**DOI:** 10.1021/jacs.6c09673

**Published:** 2026-06-22

**Authors:** Tom J. Smak, Dylan J. Walsh, Alexander Shaw, Linda J. Broadbelt, Ina Vollmer, Bert M. Weckhuysen, Shannon S. Stahl

**Affiliations:** † Inorganic Chemistry and Catalysis group, Institute for Sustainable and Circular Chemistry, Faculty of Science, 84889 Utrecht University, Universiteitsweg 99, 3584 CG Utrecht, The Netherlands; ‡ Department of Chemistry, University of Wisconsin—Madison, Madison, Wisconsin 53706, United States; § The Wisconsin Energy Institute, University of Wisconsin—Madison, Madison, Wisconsin 53726, United States; ∥ Department of Chemical and Biological Engineering, 3270 Northwestern University, Evanston, Illinois 60208, United States

## Abstract

Selective oxidation of alkanes to oxygenated products remains a fundamental challenge, particularly if the goal is to promote C–C bond cleavage while minimizing formation of CO_2_. Oxidation conditions that use O_2_ and nitrogen oxides (NO_*x*_) have been shown to be very effective in promoting radical-mediated functionalization and oxidative carbon–carbon cleavage in saturated hydrocarbon polymers, such as polyethylene. Here, we investigate the mechanism of O_2_/NO-mediated oxidation of *n-*decane as a prototypical linear alkane substrate. These reactions enable identification and quantification of reactive intermediates, including nitrites, nitrates, alcohols, and ketones. Under the reaction conditions, these species convert into common ketone intermediates that evolve into α-diketones and other α-functionalized ketones, which undergo further conversion into carboxylic acids. Infrared (IR) spectroscopy indicates that HDPE oxidation proceeds through similar key intermediates. Together, these findings establish a mechanistic framework for NO_*x*_-mediated alkane oxidation and provide a foundation for the development of broadly applicable oxidative transformations.

## Graphical abstract

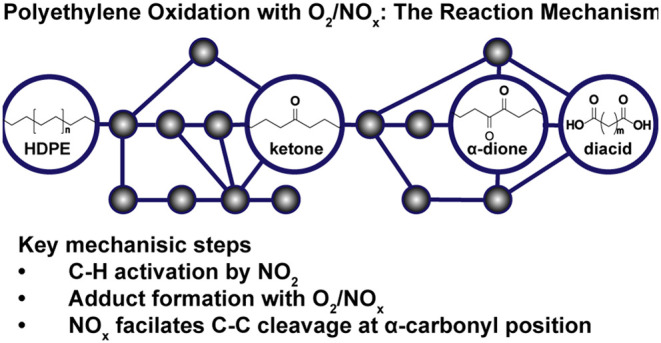

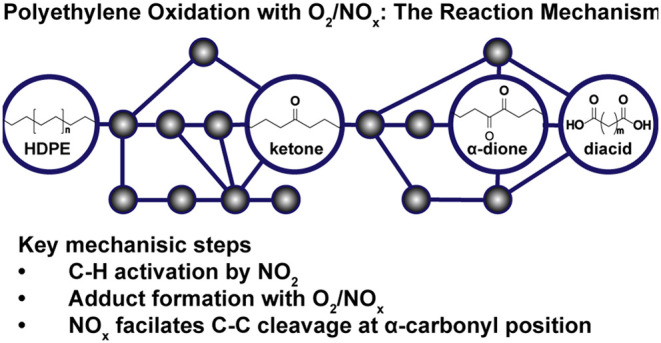

## Introduction

Selective oxidation of alkanes to oxygenated products is a longstanding challenge in chemistry, owing to the strength and ubiquity of C–H bonds and the propensity to promote full oxidation to CO_2_.^1–4^ Despite these challenges, the aerobic oxidation of alkanes is featured in prominent industrial processes, such as the oxidation of cyclohexane to cyclohexanol and cyclohexanone (K/A oil) and the Mid-Century process for the production of terephthalic acid. These processes typically proceed through complex radical-chain mechanisms involving oxygen and, in many cases, NO_*x*_ or metal-based cocatalysts. Detailed elucidation of these mechanisms—including reaction pathways, catalyst speciation, and the molecular origins of rate and selectivity—has been crucial to their development, optimization, and industrial success.^5^

More recently, there has been growing interest in extending alkane oxidation methods to the valorization of other hydrocarbon materials, including polyolefins such as polyethylene (PE), the world’s most widely produced synthetic polymer.^6^ Chemical recycling strategies that convert PE into smaller, value-added molecules have attracted increasing attention.^7^ Among these, oxidative methods are particularly appealing because they are thermodynamically favorable, can operate under relatively mild conditions, and generate oxygenated products that are directly compatible with existing chemical value chains (Figure 1a).^8–14^ In 1998, Sen and co-workers demonstrated that an O_2_/NO gas mixture could be used to convert PE into a mixture of dicarboxylic acids (C4 – C7) in yields up to 39 mol %.^15,16^ The products included industrial chemicals, such as succinic and adipic acid. Several years later, Partenheimer generated a similar mixture of dicarboxylic acids by oxidizing PE under conditions analogous to those used to produce terephthalic acid in the Mid-Century process (Co/Mn/Br in acetic acid with O_2_).^17^ More recently, these oxidative processes have been adapted, with the goal of converting complex product mixtures into valuable products. For example, early stage commercial efforts by Novoloop used HNO_3_ as a stoichiometric oxidant to convert PE into dicarboxylic acids in a process related to the O_2_/NO oxidation conditions of Sen.^18^ These products are used as building blocks for polyurethane materials.^12^ In other efforts, oxygenation product mixtures have been used as bioavailable feedstocks for microbial funneling to produce pharmaceuticals^19^ or polyhydroxyalkanoates.^10^

**Figure 1:**
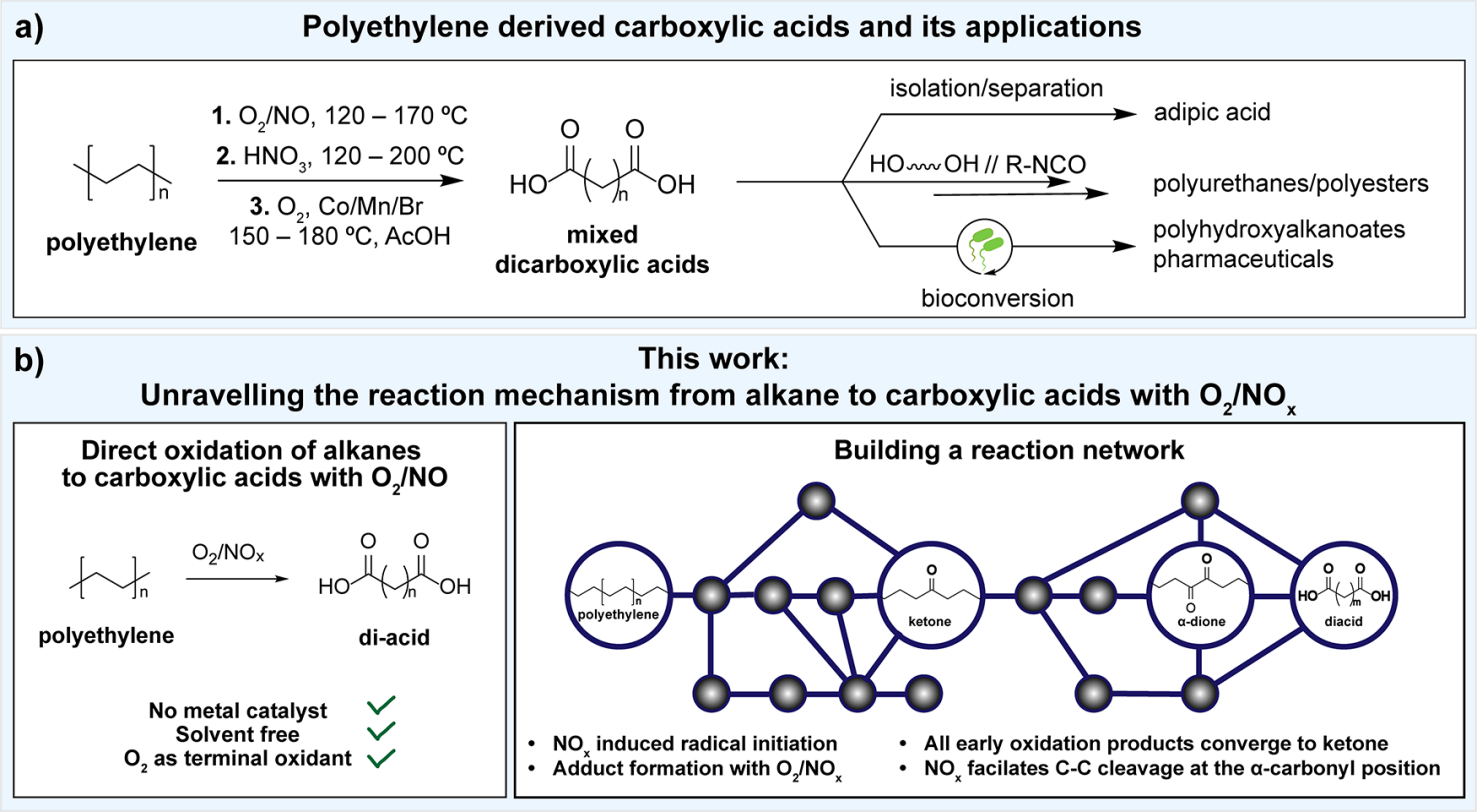
(a) Three representative conditions that promote oxidative conversion of polyethylene (PE) into dicarboxylic acids (C4 – C8), which can then be used to access valuable products. (b) This work, focused on the reaction mechanism of O_2_/NO-promoted oxidation of linear alkanes to produce dicarboxylic acids (left), elucidates a reaction network with multiple intermediates that progress through ketones and α-functionalized ketones in the formation of dicarboxylic acids (right).

The O_2_/NO conditions of Sen are noteworthy because they are solvent-free, achieve high carbon recovery from PE, and rely on catalytic cycling of NO_*x*_ species while using O_2_ as the terminal oxidant (Figure 1b). In spite of the favorable features of this process, the mechanism of O_2_/NO-promoted conversion of linear alkanes into low molecular weight products has not been investigated. The most relevant precedents are studies performed multiple decades ago exploring PE stability in the presence of O_2_, NO_2_ and HNO_3_.^20–23^ In the present study, we explore the oxidation of *n-*decane, a prototypical linear alkane that facilitates thorough product characterization and time-course analysis using gas chromatography–mass spectrometry (GC-MS). The data indicate parallel formation of alcohols and organic nitro, nitrite, and nitrate species, all of which converge into ketone intermediates. The ketone then undergoes α-functionalization to afford another series of intermediates that lead to C–C cleavage and, ultimately, carboxylic acid formation. Preliminary analysis of HDPE oxidation under similar conditions by infrared (IR) spectroscopy is consistent with an oxidative reaction pathway similar to that of decane. Collectively, the results provide the basis for a reaction network underlying the efficient conversion of linear alkanes into carboxylic acids in the presence of O_2_/NO (Figure 1b) and lay a foundation for further optimization of oxidative transformations of linear hydrocarbons.

## Results and Discussion

### O_2_/NO Oxidation of Decane and the Reactivity of Early Intermediates

*n*-Decane was selected as the substrate for mechanistic studies because it is a good model compound for HDPE and its oxidation products can be identified and quantified with high precision by using GC-MS and flame ionization detection (GC-MS/FID) or isolated and fully characterized. We initiated our investigation into the oxidation of decane with O_2_/NO by conducting a time-resolved experiment to identify reaction products and intermediates (note: detailed safety considerations associated with these experiments are outlined in section 2 of the Supporting Information (SI)). In the experiments, 10 mL of decane was loaded into a heavy walled glass reactor, and oxygen was introduced into the reactor at 15 mL/min with a backflow pressure of 100 psi. The reactor was then heated to 140 °C using an oil bath, followed by the introduction of a NO flow of approximately 25 mL/min (20% in N_2_). Upon contact of NO with O_2_, there was an immediate formation of an orange gas indicative of NO_2_, which exists in equilibrium with its dimer, N_2_O_4_.^24^ Other NO_*x*_ species, including HNO_2_ and HNO_3_, are expected to form *in situ* under these reaction conditions.^24–26^ The reaction mixture was analyzed at multiple time points by GC-MS/FID and IR spectroscopy. For the GC analysis, each sample was treated with *N*-methyl-*N*-(trimethylsilyl)­trifluoroacetamide to convert protic functional groups, such as carboxylic acids and alcohols, to their corresponding trimethylsilyl (TMS) derivatives. The injection temperature was adjusted to 200 °C to avoid decomposition of thermally unstable products, such as nitrites and nitrates.

The compounds formed in the reaction include ketones, alcohols, carboxylic acids, and alkyl nitrites, nitrates, and nitro compounds (Figure 2a). The earliest functionalized species observed were secondary nitrites, nitrates, and nitro compounds (Figure 2d). These appeared as groups of four peaks with similar intensity, suggesting a uniform degree of functionalization at the four different secondary positions. Products arising from oxidation at primary positions were not detected (Figures 2b and S2). As the reaction progressed, alcohols and ketones formed, followed by carboxylic acids. As the reaction continued, the concentration of carboxylic acids increased steadily, while secondary nitro groups accumulated and plateaued at ∼35 mol %. Other, more reactive intermediates, including alcohols, ketones, and alkyl nitrites remained low (up to 3 mol %) throughout the reaction. When the NO partial pressure was reduced to achieve an O_2_/NO ratio of ≥10:1, organic nitrates became the dominant nitrogen-containing species with minimal nitrite and nitro formation (Figure 2e). These conditions also led to an overall decrease in oxidation levels, with the decane conversion dropping from 17 mol % after 1 h under the standard condition to 3 mol % at the low NO conditions. Meanwhile, experiments conducted with NO or O_2_ alone led to minimal conversion under otherwise identical conditions (SI, Figure S1b,c).

**Figure 2:**
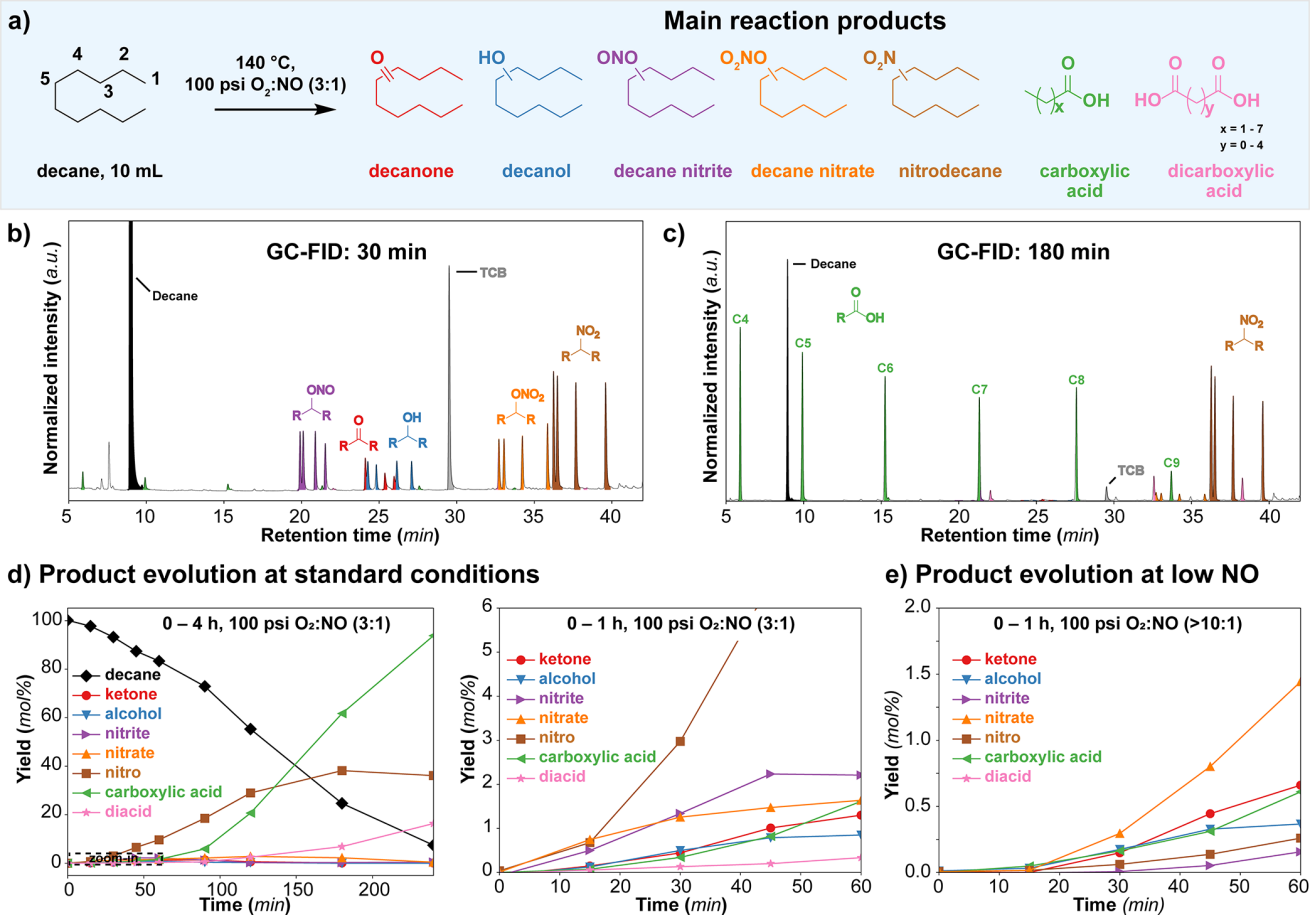
(a) Major products detected from the oxidation of decane under standard conditions (*i.e*., 140 °C, 100 psi, O_2_ flow = 15 mL/min, NO/N_2_ flow (20% NO in N_2_) = 25 mL/min). (b) Gas chromatography-flame ionization detection (GC-FID) data at 30 min. All identified peaks are color-coded; the gray peak is the internal standard 1,2,3 trichlorobenzene (TCB). (c) GC-FID data recorded at 3 h. (d) Product evolution as a function of time under standard conditions (see caption part “a”) (e) Product evolution as a function of time with low NO pressure (*i.e*., 140 °C, 100 psi, O_2_ flow = 15 mL/min, NO/N_2_ flow (20% NO in N_2_) < 2.5 mL/min).

To gain deeper insights into the reaction mechanism, a series of experiments were conducted to probe the fate of early oxidation products under relevant reaction conditions (Figure 3). Various secondary nitrite, nitrate, nitro, oxime, alcohol, and hydroperoxide compounds were synthesized or purchased, and their reactivities were analyzed in one of two ways. For intermediates expected or observed to be more reactive (alcohol, nitrite, oxime and hydroperoxide), the O_2_/NO oxidation experiments were performed at lower temperature (80 or 100 °C) in cyclohexane. Performing reactions in cyclohexane simplifies product analysis by avoiding the formation of decane-derived background products, while still providing a reaction environment comparable to neat decane. This reduction in background ensures that peaks from the additives and their derivatives are easily distinguishable, making the data analysis more straightforward and reliable. The reactivity of less reactive nitrate and nitro compounds was analyzed in ″spiking experiments″ in which ∼1% of the species of interest was added to the standard conditions to assess the impact on the formation of other intermediates.

**Figure 3:**
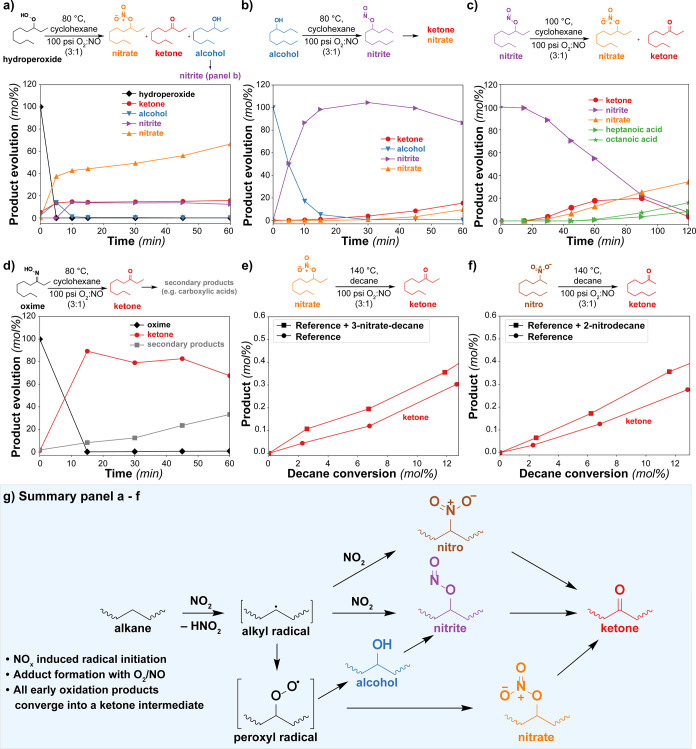
Experiments probing the fate of several proposed early stage reaction intermediates. All experiments were executed at a pressure of 100 psi (O_2_ flow = 15 mL/min, NO/N_2_ flow (20% NO in N_2_) = 25 mL/min). (a) Experiment with decane-3-hydroperoxide (80 °C in cyclohexane). (b) Experiment with 4-decanol (80 °C in cyclohexane). (c) Experiment with decane-3-nitrite (100 °C in cyclohexane). (d) Experiment with 3-decanone oxime (80 °C in cyclohexane). (e) Experiment with added decane-3-nitrate (140 °C in decane). (f) Experiment with added 2-nitrodecane (140 °C in decane). (g) A proposed reaction scheme rationalizing the observation of experiments shown in Figures 2 and 3a–f.

The reaction of decane 3-hydroperoxide in cyclohexane led to near immediate conversion to 3-decyl nitrate, in addition to the smaller quantities of the alcohol and ketone (Figure 3a), while 4-decanol underwent quantitative conversion to the secondary nitrite in approximately 15 min under the same reaction conditions (Figure 3b). A secondary decyl nitrite converted into mixture of decyl nitrate and decanone in similar quantities over a longer reaction time (∼90 min; Figure 3c). Oximes were not detected in the decane oxidation experiments in Figure 2, but they represent possible intermediates in NO_*x*_-mediated oxidations.^27^ Analysis of the reactivity of 3-decyl oxime showed complete conversion by the first time point, forming the corresponding ketone as the major product, together with carboxylic acids and other secondary oxidation products that increase slowly at longer reaction times (Figure 3d). This rapid reactivity suggests that oximes will not be observed if they form as intermediates in the reaction. Finally, spiking experiments were conducted with 3-decyl nitrate (Figure 3e) and 2-nitrodecane (Figure 3f) at 140 °C in decane. Both compounds led to a moderate increase in the quantity of ketone product, with the amount likely limited by the intrinsically higher reactivity of the ketone relative to nitro and nitrate derivatives.

The data outlined in Figures 2 and 3a–f may be rationalized by the mechanism in Figure 3g. The reaction is expected to be initiated by hydrogen atom transfer (HAT) mediated by NO_2_, which is abundant under our reaction conditions and forms a H–ONO bond that has a bond dissociation energy 25–30 kcal/mol higher than that of the H–OO and H–NO bonds formed if O_2_ or NO is the initiator.^28–30^ The negligible reactivity observed from the control experiments involving decane reactions with NO or O_2_ alone aligns with this proposal. A secondary alkyl radical formed by HAT will undergo a nearly barrierless addition of NO_2_, NO, or O_2_.^25,31,32^ Reaction with NO_2_ can form either a N- or O-bound adduct, resulting in a nitro or nitrite species.^33^ Experimentally, we observed a nitro/nitrite ratio of ∼2 in the early stages of the reaction, consistent with previous observations of PE reactivity with NO_2_.^23^ Organic hydroperoxides, the primary intermediates observed in conventional autoxidation of alkanes,^34^ were not detected under the present reaction conditions. Any peroxyl radical formed will undergo a barrierless reaction with NO_2_ or NO^31^ leading to the formation of thermally unstable peroxynitrate (R-OONO_2_) or peroxynitrite (R-OONO) species.^24,33^ These species have weak O–O bonds that can undergo homolysis to form alkoxy radicals that can subsequently react with NO_2_ to form nitrates or promote HAT from a C–H bond to form an alcohol.^35^ The experiment performed at a higher O_2_/NO ratio (≥10:1; Figure 2e) shows a significant enhancement in the formation of nitrate intermediates, relative to nitro and nitrite intermediates, aligning with the expectation that intermediate alkyl radicals are more likely to be trapped by O_2_ at low partial pressures of NO. Alkyl nitrites and nitrates can undergo thermal decomposition into ketones, while secondary nitrodecanes are comparatively less reactive and show the highest steady-state persistence under the reaction conditions (Figure 2d). Despite the varying reactivities of the intermediates, the data suggest that most, if not all, ultimately convert to a common ketone intermediate that continues to react further. This observation motivated the next set of experiments focused on the fate of ketone intermediates.

### Reactivity of Ketones and Their Conversion into Carboxylic Acids

The convergence of early stage reaction intermediates into ketones suggests that C–C cleavage will proceed through this intermediate. To directly probe ketone reactivity, spiking experiments were performed in which a small amount of symmetric 6-undecanone was subjected to O_2_/NO oxidation in cyclohexane at 100 °C (Figure S4). Under these conditions, the major products observed were hexanoic acid and pentanoic acid in an approximately 1:1 ratio. This product distribution is consistent with selective C–C bond cleavage α to the ketone, rather than nonselective radical fragmentation pathways. To further investigate the role of the α-position in ketone oxidation, 6-undecanone-5,5,7,7-*d*_4_ was synthesized and evaluated under identical O_2_/NO oxidation conditions. Comparison of the oxidation rates of undeuterated and deuterated 6-undecanone gave an apparent kinetic isotope effect (KIE) of 3.2 (Figure S5). This magnitude is consistent with a primary kinetic isotope effect, indicating that C–H bond cleavage at the α-position is involved in, or occurs prior to, the rate-determining step.

With the α-position identified as the key reactive site, we next sought to distinguish between direct HAT and an enol-mediated oxidation pathway. To evaluate the feasibility of an enol-based mechanism, 6-undecanone and 6-undecanone-5,5,7,7-*d*_4_ were heated at 100 °C under N_2_ in a cyclohexane/acetic acid mixture (8:2 v/v; Figure S6) in an attempt to observe the rate of H/D scrambling. Acetic acid was included because acidic conditions are known to accelerate enolization, and carboxylic acids are generated in significant quantities during the oxidation reaction. Reaction progress was monitored by GC–MS by tracking the isotopic distribution of the ketone over the *m*/*z* range of 170–175 Da, corresponding to isotopologues derived from undeuterated 6-undecanone (170.17 Da) and 6-undecanone-5,5,7,7-*d*_4_ (174.19 Da). Under these conditions, only minimal enolization was observed for both the undeuterated and deuterated ketones, suggesting that acetic acid alone does not promote enolization rapidly enough for this pathway to account for ketone oxidation under the reaction conditions. However, HNO_3_ and H_2_O are also generated during O_2_/NO oxidation and may further accelerate enolization. To better mimic the oxidative environment, additional experiments were conducted in a cyclohexane/acetic acid/H_2_O/HNO_3_ mixture (8:1.5:0.4:0.1 v/v/v/v) under N_2_ at 100 °C (Figure S7). Under these conditions, significant enolization was observed on a time scale comparable to ketone oxidation. The reaction exhibited apparent zero-order kinetics and gave a KIE of 4.9. These results indicate that enolization in the presence of HNO_3_ is sufficiently rapid to be considered a viable pathway in ketone oxidation. The KIE values observed for ketone oxidation (3.2), enolization (4.9) and that expected for direct HAT do not allow for clear distinction between direct HAT and enol-mediated oxidation pathways. We conclude that both mechanisms remain plausible under the O_2_/NO reaction conditions, and this conclusion is further supported by the computational studies elaborated below.

With the role of the α-position in ketone oxidation clarified, we then sought to identify ketone oxidation intermediates. A time course oxidation of neat 3-decanone at 80 °C revealed the formation of several intermediates prior to the formation of carboxylic acids (Figure 4a). The most prominent species consisted of α-diketone, α-nitro ketone, and α-nitrate ketone species, as established by GC-MS, NMR and IR spectroscopy (Figure 4b). MS and IR spectroscopic data supported the presence of nitrates (1630/1640 cm^–1^, asym. NO_2_ stretch)^36^ and nitro groups (1560/1575 cm^–1^, asym. NO_2_).^36^ The IR peaks appear at slightly higher energies than those of secondary alkyl nitrates and nitro compounds without an adjacent ketone.^36^ ^1^H NMR spectroscopic data revealed peaks consistent with an α-nitrate (6.16/6.30 ppm) and α-nitro (5.14/5.25 ppm) ketones,^37^ each exhibiting a quartet for the methine proton adjacent to a methyl group and a triplet (nitrate) or AB quartet (nitro) for the methine adjacent to a methylene. The similar peak intensities indicate that functionalization occurred with nearly equal probability on each side of the ketone. The resonances exhibit chemical shifts ∼1 ppm higher than resonances for secondary alkyl nitrates and nitros that lack an adjacent ketone.^38,39^ In addition to the three major intermediates, MS fragmentation patterns and NMR data support the presence of trace quantities of ketones with an α-oxime group (SI, Section 5).

**Figure 4:**
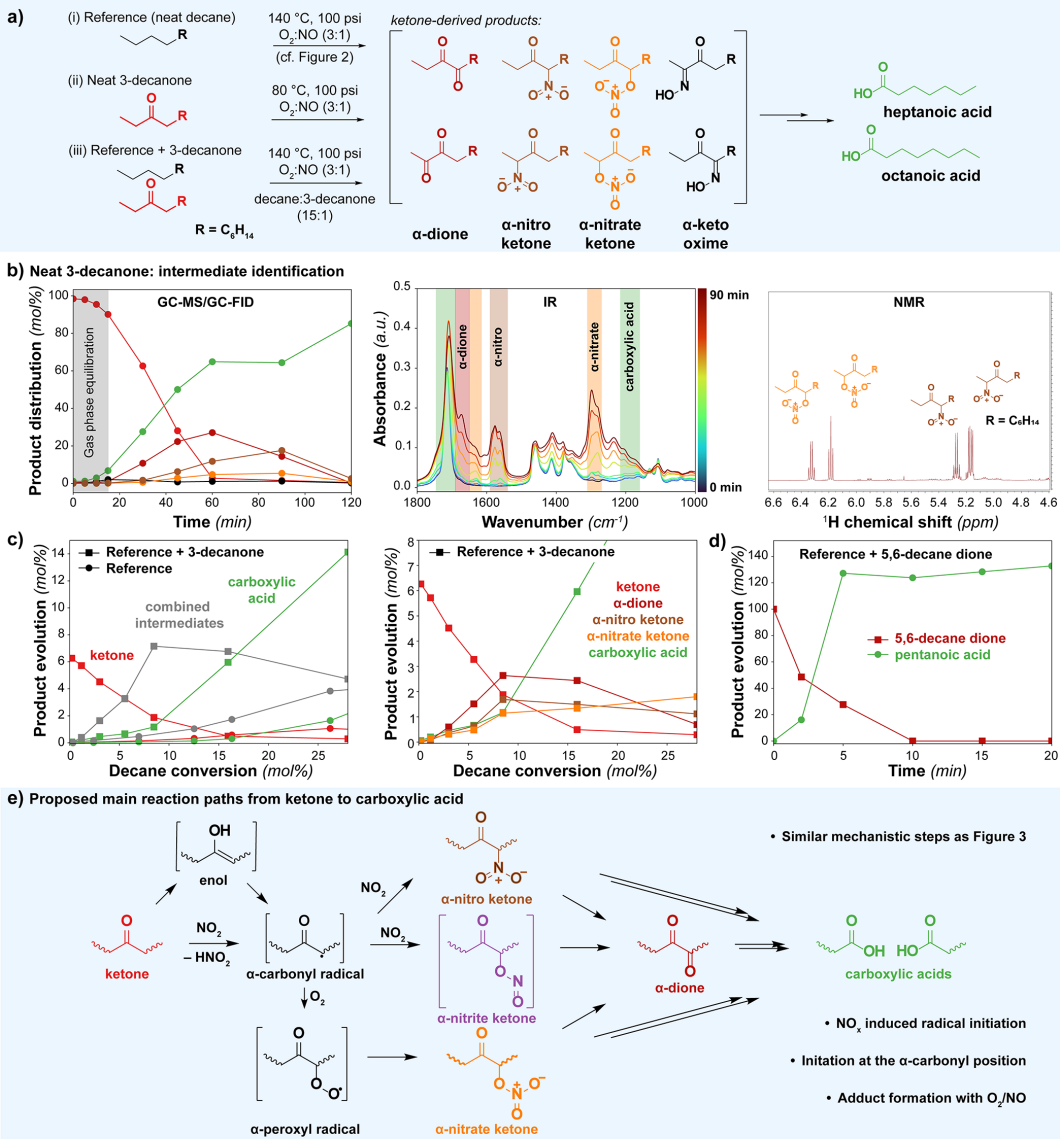
(a) Three different reaction conditions evaluating the formation and/or conversion of 3-decanone during O_2_/NO oxidation conditions, in addition to the identities of observed intermediates and final products. (b) Time-course data and IR/^1^H NMR spectroscopic data from the oxidation of neat 3-decanone. Conditions: 80 °C, 100 psi, O_2_ flow = 15 mL/min, NO flow (20% in N_2_) = 25 mL/min. (c) Time-course data from a standard oxidation of decane (“Reference”) and a spiking experiment with 3-decanone (“Reference + 3 decanone”). Conditions: 140 °C, 100 psi, O_2_ flow = 15 mL/min, NO flow (20% in N_2_) = 25 mL/min. (d) Time-course data from a spiking experiment with 5,6-decane dione (“Reference +5,6-decane dione”). Conditions: 140 °C, 100 psi, O_2_ flow = 15 mL/min, NO flow (20% in N_2_) = 25 mL/min. (e) Proposed reaction path for the conversion of ketones into carboxylic acids.

The same α-diketone, α-nitro ketone, and α-nitrate ketone products were identified in the spiking experiment, performed by adding 3-decanone (6.7 mol %) to the reaction of decane at 140 °C (Figure 4c). Initial growth of these intermediates at early stages of the reaction was followed by slow decay, together with formation of heptanoic and octanoic acids. An independent spiking experiment performed with 5,6-decanedione revealed rapid conversion of this species into pentanoic acid (Figure 4d). The reactivity of α-nitro and α-nitrate ketones were not independently tested, but the conversion of nitroalkanes and alkyl nitrates into ketones outlined above (cf. Figure 3) suggests that these intermediates could be converted to the diketone and progress further into carboxylic acid products. A spiking experiment conducted with an α-keto oxime, decane-2-one-3-oxime, led to parallel formation of octanitrile and octanoic acid (SI, Figure S3a); however, a control experiment showed that nitriles do not react further under standard reaction condition (SI, Figure S3b). These observations, together with the lack of significant nitrile formation during decane oxidation, suggest that α-keto oximes are not major reaction intermediates under the O_2_/NO conditions.

These observations indicate that the formation of carboxylic acids from ketones is initiated by a second HAT at the C–H bond adjacent to the carbonyl group of the ketone or through an enolization-mediated pathway. The resulting intermediates can subsequently react with NO_2_ or O_2_, leading to the formation of α-nitro, α-nitrite, and α-peroxycarbonyl species (Figure 4e). These species can then progress through steps analogous to those outlined in Figure 3g, resulting in formation of an α-diketone that can rapidly undergo C–C cleavage and formation of carboxylic acids. Direct oxidative C–C cleavage from the α-nitro and α-nitrite ketones is also possible. The experimental data indicate that decanones react more rapidly than decane, evident from the minimal buildup of decanones as intermediates in Figure 2d and from the rapid conversion of 3-decanone at a temperature 60 °C lower than the standard decane reaction conditions (cf. Figure 4b). This enhanced reactivity is attributed to the much weaker bond strength of C–H bonds adjacent to ketones [395 kJ/mol (α-ketone) vs 415 kJ/mol (secondary alkyl)^40^], which will lead to much faster reactivity with NO_2_ and other reactive radicals in the reaction mixture.^41^

### Computational Analysis of Reaction Energetics in O_2_/NO-Mediated Oxidation of *n*-Alkanes

Density functional theory (DFT) calculations were performed to evaluate the energetics of key steps in the O_2_/NO-mediated oxidation of *n*-alkanes to assess the feasibility of the proposed radical pathways. Geometry optimizations and frequency calculations for intermediates and transition states were performed at the M06–2X/aug-cc-pVTZ^42–44^ level using the PCM continuum model with *n*-decane as the solvent; open-shell species were treated using unrestricted DFT. Gibbs free energies (140 °C), including solution-phase entropy corrections, were computed using *n*-hexane as a representative substrate. Figure 5a summarizes pathways for oxidation of *n*-hexane to 3-hexanone, while Figure 5b outlines subsequent oxidation of 3-hexanone to 3,4-hexanedione.

**Figure 5:**
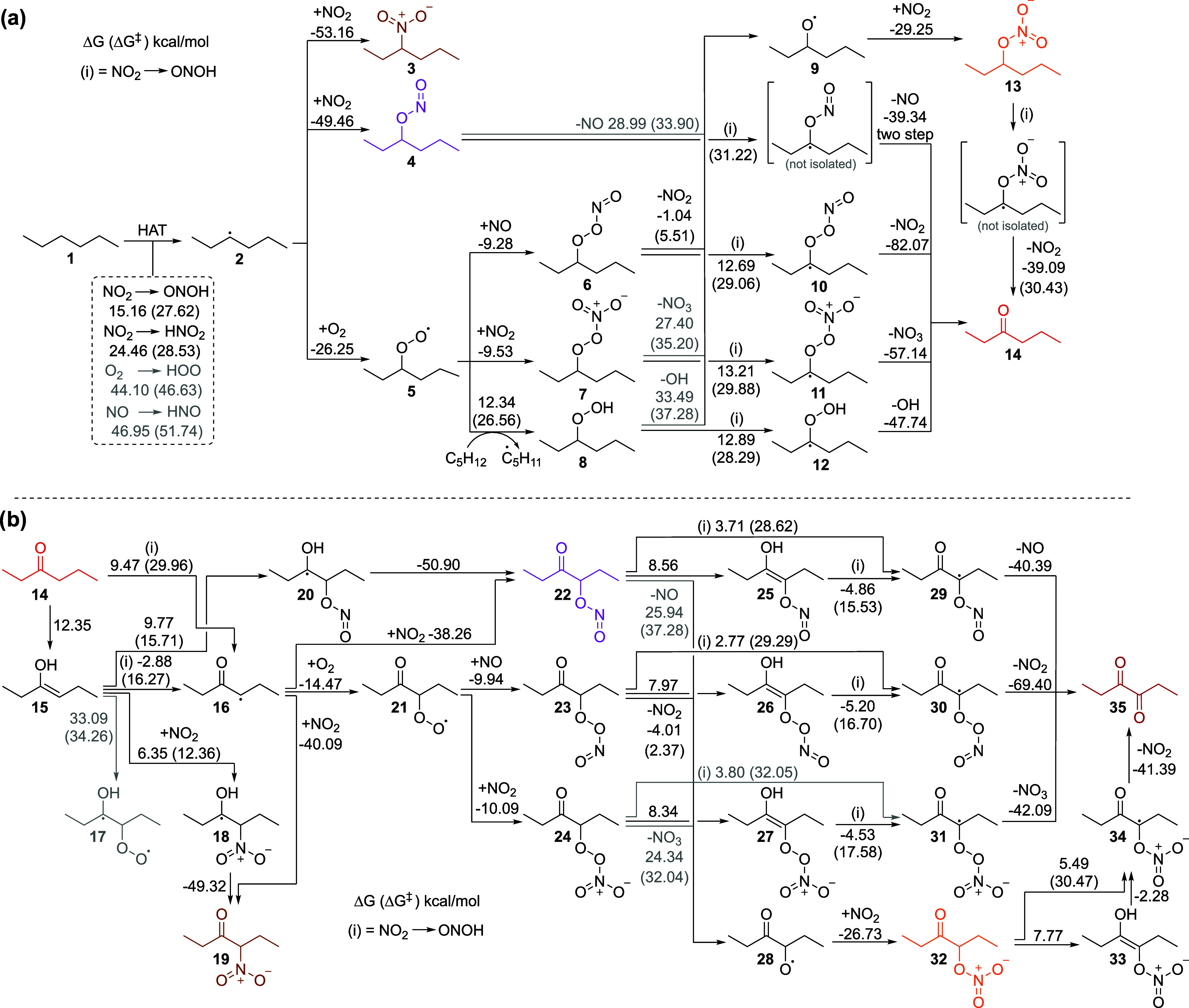
(a) DFT calculations of relative free energies for intermediates and transition states along representative pathways for the oxidation of *n*-hexane to 3-hexanone at 140 °C. (b) DFT calculations of relative free energies for pathways for the oxidation of 3-hexanone to 3,4-hexanedione at 140 °C. Energies are reported in kcal·mol^–1^, with intermediate free energies given without parentheses and transition-state energies in parentheses. Species labeled “not isolated” correspond to transition states that could not be identified. (i) signifies the use of NO_2_ radical for HAT.

Initial C–H activation was evaluated for several oxidants present under the reaction conditions, including NO_2_ (via N and O), NO, and O_2_ (Figure 5a). Among these, NO_2_-mediated HAT is most favorable, with N- and O-centered pathways exhibiting comparable activation free energies, but O–H bond formation is less endergonic. The HAT reactions with NO or O_2_ show much higher barriers and significantly less favorable thermodynamics. Given these results, NO_2_ (with O–H bond formation) was used in subsequent calculations involving HAT. The alkyl radicals generated by HAT are readily intercepted by NO_2_ or O_2_, in very favorable (Δ*G*° = −26 – −53 kcal/mol), and likely diffusion controlled, reactions. These pathways lead to formation of nitro (**3**) and nitrite (**4**) intermediates, consistent with experiment (Figure 2), as well as a peroxyl radical (**5**). From the peroxyl radical (**5**), several downstream pathways are accessible, including hydrogen atom abstraction (Δ*G* ^‡^ = 26.6 kcal·mol^–1^) to form hydroperoxide **8** or trapping by NO_2_ or NO to give intermediates **7** and **6**, respectively. Intermediates **6**–**8** can undergo α-C–H abstraction and elimination of NO_2_, NO_3_, or OH radicals to yield 3-hexanone (**14**), with calculated barriers (≈28–30 kcal·mol^–1^) consistent with overall exergonic profiles. Pathways involving alkoxyl radical formation (**9**) were also examined; however, these are generally associated with higher barriers, with only the NO-derived pathway (**6** → **9**) exhibiting a comparatively low barrier. Conversion of nitrate intermediates (**13**) to ketones proceeds through higher barriers (≈ 30 kcal·mol^–1^) that should be feasible at elevated temperatures, consistent with experimental observations (Figure 3e).

Figure 5b depicts oxidation of 3-hexanone (**14**) to 3,4-hexanedione (**35**). Two pathways for α-functionalization were considered: direct α-C–H abstraction and enolization followed by HAT. Direct HAT proceeds with a relatively high, but accessible, barrier at the 140 °C (Δ*G* ^‡^ ≈ 30 kcal·mol^–1^) to generate a carbon-centered radical (**16**). Enolization is thermodynamically uphill but enables lower-barrier HAT from the O–H bond (Δ*G* ^‡^ ≈ 16 kcal·mol^–1^ from enol **15**). The resulting resonance-stabilized radical (**16**) can be trapped at the carbon center by NO_2_ or NO, or alternatively by O_2_ to form peroxyl radical **21**, which can be further intercepted by NO or NO_2_ to yield intermediates **23** and **24**. An alternative pathway proceeds directly from the enol (**15**) via radical addition of NO_2_ to the distal alkene carbon, which occurs with low barriers to form keto-nitro (**19**) and keto-nitrite (**22**) species, providing products analogous to those formed via HAT pathways. Subsequent transformations of α-functionalized intermediates (**22**–**24**) follow pathways similar to those described above, with enolization and HAT steps remaining energetically accessible. Importantly, all intermediates examined exhibit viable pathways to the α-diketone, supporting its role as a key precursor to C–C bond cleavage.

Overall, the DFT results reveal multiple kinetically accessible pathways involving hydrogen atom transfer (HAT), radical trapping, and subsequent functionalization. A key insight is that NO_2_ serves as the primary initiator of the oxidation process and also facilitates propagation, while not being significantly incorporated into the final products, which primarily consist of carboxylic acids. NO_2_ significantly accelerates the overall rate relative to conventional autoxidation while maintaining analogous mechanistic pathways and yielding similar final products (especially, carboxylic acids). This interconnected reaction network enables efficient conversion of hydrocarbons to ketones, α-dicarbonyl compounds, and ultimately carboxylic acids under O_2_/NO conditions.

### O_2_/NO-Mediated Oxidation of High-Density Polyethylene

The O_2_/NO mediated oxidation conditions were then adapted to reactions with HDPE (*M*_n_ = 16 010 g/mol and *M*_w_ = 137 500 g/mol). The reactions were performed in a Parr autoclave with a static pressure of O_2_ and NO diluted with N_2_ (120 °C, *P*_NO_ = 4 bar, *P*_O_2__ = 8 bar, *P*_N_2__ = 28 bar), and the reaction progress was ended at several different time periods, from 0.5–5 h, to analyze the product by GC-MS/FID, IR spectroscopy, and CHN analysis. The oxidized product typically consisted of a colorless-to-yellow oil, with a mass increase of up to 80% relative to the initial polymer, reflecting incorporation of oxygen and nitrogen atoms into the material. The GC-MS/FID data revealed that C4–C8 dicarboxylic acids as the primary low molecular weight reaction products (Figure 6a), and they are supplemented by small quantities of dicarboxylic acids bearing a secondary nitro group. The observed chain lengths are comparable to those reported for the HNO_3_-based systems described previously by Novoloop,^18^ but they are shorter than those typically obtained under Mid-Century oxidation conditions,^17^ which generate diacids with broader molecular weight distributions that extend to higher molecular weights. The carbon recovery of the oil fraction, determined by CHN elemental analysis, was excellent (>80%) over the first 2 h, but gradually decreased at longer reaction times (Figure 6b). The dicarboxylic acid yield steadily increased from 1–5 h, reaching approximately 29 mol % at 5 h. The unrecovered carbon is assumed to be present in gaseous products (*e.g*., CO/CO_2_), possibly resulting from the decomposition of malonic acid^45^ or from unselective C–C bond cleavage of α-derivatized ketone species.

**Figure 6:**
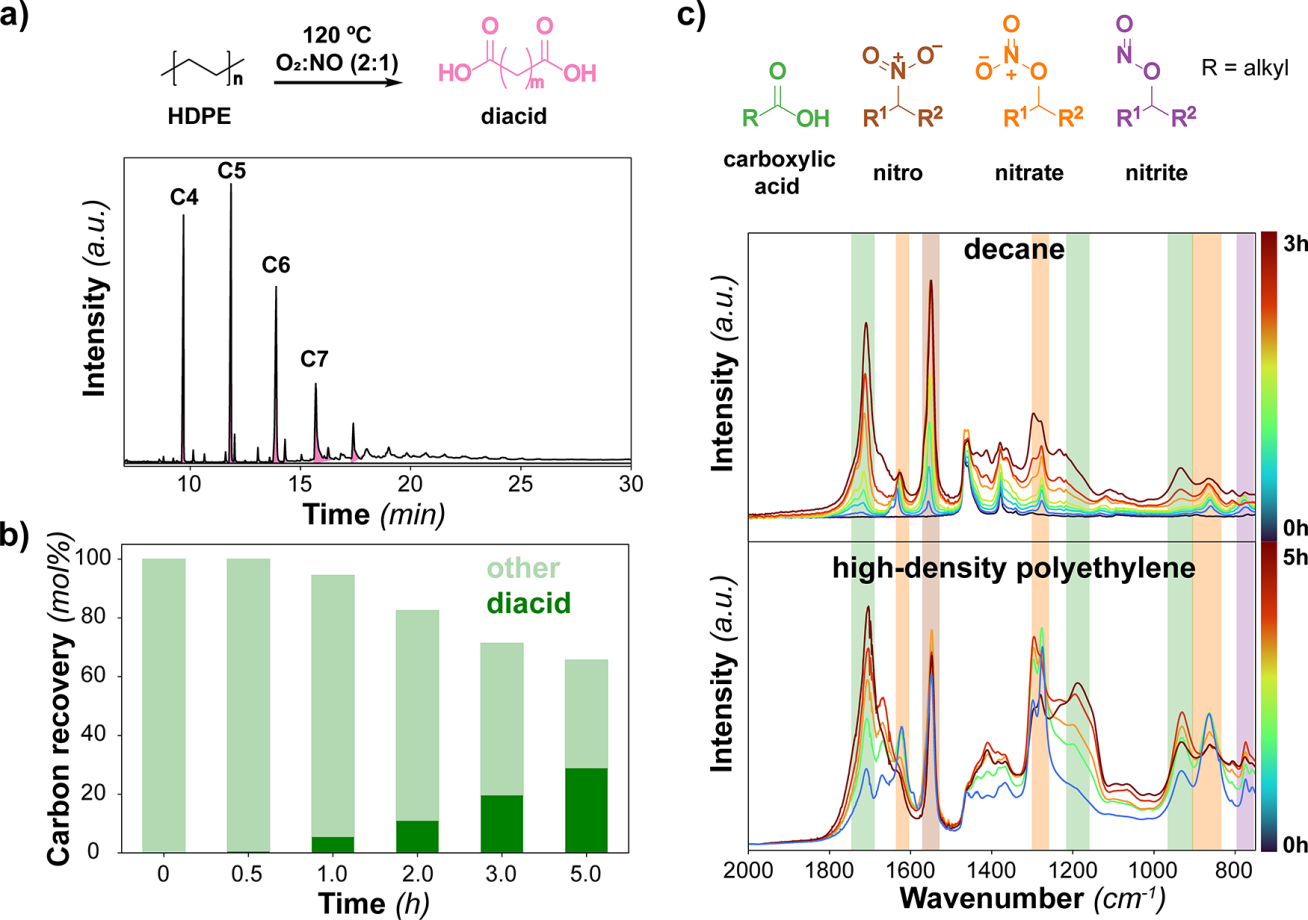
(a) Gas chromatography-flame ionization detection (GC-FID) data of a polyethylene (PE) oxidation experiment showing the formation of dicarboxylic acids (120 °C, 5 h, *P*_O_2__ = 8 bar, *P*_NO_ = 4 bar, *P*_N_2__ = 28 bar). (b) The dicarboxylic acid yield and carbon balance obtained with elemental (CHN) analysis as a function of time (120 °C, *P*_O_2__ = 8 bar, *P*_NO_ = 4 bar, *P*_N_2__ = 28 bar). (c) A comparison of IR data between decane (140 °C, 100 psi, O_2_ flow = 15 mL/min, NO flow (20% in N_2_) = 25 mL/min) and HDPE (120 °C, *P*_O_2__ = 8 bar, *P*_NO_ = 4 bar, *P*_N_2__ = 28 bar) oxidation as a function of time.

The yield is defined as the percentage of carbon atoms from the original PE that ends up in the dicarboxylic acid product. IR spectroscopy was performed on the full reaction mixture to analyze all species in the oil, including those not detected by GC-MS/FID. IR data obtained from product mixtures from decane and PE oxidation (Figure 6c) revealed strong similarities, consistent with similar reaction pathways with these low and high molecular weight substrates. The main peaks evident from both reactions arise from the presence of carboxylic acid (1710, 1240, 940 cm^–1^), nitrate (1625, 1280, 870 cm^–1^), and nitro groups (1550 cm^–1^). A small quantity of nitrite groups (780 cm^–1^) were also detected at early stages of the decane oxidation. The functional groups and their relative intensities are readily rationalized by the products identified from decane oxidation on the basis of GC-MS/FID data (cf. Figures 2–4). This correlation suggests that mechanistic insights gained from decane oxidation are directly applicable to reactions with polymeric samples.

### Relationship to Other NO_*x*_-Based Oxidation Reactions

The lack of mechanistic analysis of O_2_/NO-mediated oxidation of linear alkanes in the literature is notable, considering the importance of alkane oxidation in industry. Among the most important industrial processes relevant to the present study is the commercial oxidation of cyclohexyl ketone and alcohol (“K/A oil”) to produce adipic acid (Figure 7). This process uses stoichiometric HNO_3_ as the oxidant, and it is performed on a global scale exceeding 4 million tons annually.^2,46,47^ In addition, there are numerous smaller scale applications and fundamental studies of NO_*x*_-mediated or NO_*x*_/O_2_-based catalytic methods for the oxidation of alcohols, ketones, and olefins.^48–51^ Each of these reactions is expected to generate a complex equilibrium of NO_*x*_-based species that could include NO, NO^+^, NO_2_, N_2_O_4_, N_2_O_3_, HNO_2_, HNO_3_, among others, with the precise speciation varying under different reaction conditions.^24^

**Figure 7:**
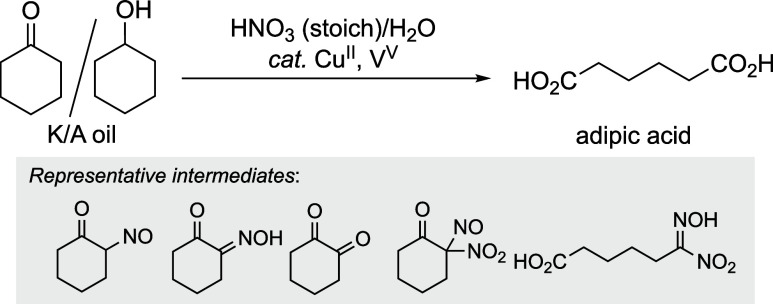
Industrial oxidation of K/A oil proceeds through multiple intermediates. Cu^II^ and V^V^ catalysts have been shown to improve the selectivity of the diketone intermediate into adipic acid.

The mechanisms of these NO_*x*_-based reactions are often not well understood, and detailed analysis is beyond the scope of this study. Nonetheless, it is worth noting that many of the reactions, including the oxidation of K/A oil to adipic acid, are performed in the presence of strong acid and form NO^+^ as a reactive oxidant through acid-promoted dehydration of HNO_2_. NO^+^ is electrophilic, and it efficiently oxidizes ketones to α-nitrosyl ketones and the isomeric oxime.^26,46,50^ Oximes do not appear to be a viable intermediate in our process (see above and Section 1.3 and Figure S3 in the SI), and the different reactivity may be rationalized by the involvement of a radical, rather than electrophilic, oxidation pathway in the present system. On the other hand, in spite of the different mechanisms, K/A oil oxidation by HNO_3_ ^46,52^ and the present linear alkane oxidation by O_2_/NO are proposed to include α-diketones as prominent intermediates. This similarity is noteworthy because oxidative cleavage of the cyclic α-diketone to adipic acid has been shown to exhibit higher selectivity (with reduced carbon loss to shorter-chain diacids) in the presence of Cu^2+^ and V^5+^ catalysts.^48,52^ This observation highlights opportunities for further improvements in the process for oxidative conversion of HDPE to diacids, suggesting that cocatalysts could support improved carbon balance and higher selectivity and yields of the target diacids (cf. Figure 6b).

## Conclusion

The O_2_/NO-mediated oxidation pathways of linear alkanes elucidated herein highlight the multifaceted roles of NO_*x*_ and O_2_ in the reaction mechanism. This understanding was achieved through systematic additive studies that tracked the fate of key intermediates, combined with comprehensive product analysis. Activation of C–H bonds via HAT to afford alkyl radicals is followed by rapid reaction with NO_2_ or O_2_ to generate nitrite, nitro, and nitrate species. These early oxidation products ultimately converge to form ketones. NO_*x*_-promoted oxidation of the ketone or its enol isomer gives rise to α-diones, α-nitro ketones, and α-nitrate ketones, which further oxidize to carboxylic acids. IR spectroscopic data from O_2_/NO-mediated HDPE oxidation reveal a similar set of intermediate species, indicating that both decane and HDPE undergo oxidation by analogous mechanisms. The insights clarify mechanistic pathways for C–C cleavage in alkanes during autoxidation, in addition to highlighting the unique ability of NO_2_ to enhance the rate of alkane autoxidation. The significantly higher O–H and N–H bond strengths in HONO and HNO_2_ relative to the O–H bond strength of HOO lead to much faster initiation and propagation steps involving HAT. Collectively, this work establishes a foundation for future efforts targeting more selective alkane oxidation methods that leverage the higher reactivity.

## Supplementary Material





## Data Availability

All data and python scripts utilized in the manuscript have been uploaded to the YODA repository and are available under 10.24416/UU01-M0MZPN.

## Notes

Experimental procedures, supplementary notes, full characterization data (including NMR spectra), and DFT thermodynamic parameters (H, S, and G for all structures) (PDF)
Cartesian coordinates (*XYZ*) for all DFT-optimized structures (TXT)

## Abbreviations

HDPE: high-density polyethylene
IR: infrared
NMR: nuclear magnetic resonance
GC-MS: gas chromatography–mass spectrometry
GC-FID: gas chromatography-flame ionization detection
SI: Supporting Information
