# Supplementary material for: Mechanism of O2/NO-Promoted Oxidative C–C Bond Cleavage in Linear Alkanes
Source: J Am Chem Soc. 2026 Jun 22;148(26):27900–11. doi: 10.1021/jacs.6c09673 (PMC13352626; doi:10.1021/jacs.6c09673)
Supplement: Supplementary file 1 [file ja6c09673_si_001.pdf]

Supporting Information

Mechanism of O_2/NO-Promoted Oxidative C–C Bond Cleavage in Linear Alkanes

Tom J. Smak,[a,b,c] *Dylan J. Walsh,*[b,c] Alexander Shaw,[d*] *Linda J. Broadbelt,*[d,] *Ina Vollmer,*[a] *Bert M. Weckhuysen,*[a,*] *and Shannon S. Stahl*[b,c,*]

[a]Inorganic Chemistry and Catalysis group, Institute for Sustainable and Circular Chemistry, Faculty of Science, Utrecht University, Universiteitsweg 99, 3584 CG Utrecht, The Netherlands

[b]Department of Chemistry, University of Wisconsin−Madison, Madison, Wisconsin 53706, United States

[c]The Wisconsin Energy Institute, University of Wisconsin−Madison, Madison, Wisconsin 53726, United States

[d]Department of Chemical and Biological Engineering, Northwestern University, Evanston, IL 60208, United States

* alexander.shaw@northwestern.edu, b.m.weckhuysen@uu.nl, stahl@chem.wisc.edu

Table of Contents

1. SUPPORTING FIGURES S3

1.1 GC TIME PROFILES OF DECANE OXIDATION USING O_2-ONLY AND NO-ONLY CONDITIONS S3
1.2 FUNCTIONAL GROUP DISTRIBUTION DURING DECANE OXIDATION S4
1.3 ANALYSIS OF C–C CLEAVAGE OF KETONE INTERMEDIATES S5

2. GENERAL EXPERIMENTAL S10

2.1 MATERIALS AND REAGENTS S10
2.2 EQUIPMENT AND INSTRUMENTATION S11
2.3 SAFETY CONSIDERATIONS S12

3. SYNTHESIS OF MODEL COMPOUNDS S13

3.1 SYNTHESIS OF 3-DECANONE OXIME S13
3.2 SYNTHESIS OF 2-NITRO DECANE S13
3.3 SYNTHESIS OF DECANE-3-NITRATE S14
3.4 SYNTHESIS OF 3-DECANYL NITRITE S14
3.5 SYNTHESIS OF 2-DECANONE-3-OXIME S15
3.6 SYNTHESIS OF DECANE-3-HYDROPEROXIDE S15
3.7 SYNTHESIS OF 5,6-DECANE DIONE S16
3.8 SYNTHESIS OF 6-UNDECANONE-5,5,7,7-D_4 .... S16

4. OXIDATION EXPERIMENTS S18

4.1 DECANE OXIDATION EXPERIMENTS S18
4.2 DECANE GC-MS ANALYSIS S18
4.3 POLYETHYLENE OXIDATION EXPERIMENTS S19
4.4 POLYETHYLENE PRODUCT ANALYSIS S19

5. NMR, IR, AND MS SPECTRA S20

6. DENSITY FUNCTIONAL THEORY (DFT) CALCULATIONS S31

7. RADICAL INHIBITION EXPERIMENT WITH BUTYLATED HYDROXY TOLUENE S34

8. GPC MEASUREMENTS S36

9. REFERENCES S37

1. SUPPORTING FIGURES

1.1 GC time profiles of decane oxidation using O_2-only and NO-only conditions

Decane oxidation experiments were performed at 140 °C with NO and O_2 only. Under NO atmosphere approximately ~2% decane conversion was observed after 4 h reaction time **(Figure S1b)**. The reactivity can most likely be ascribed to NO_2 impurities, which are known to be present in NO gas mixtures. In the absence of NO, the decane conversion is minimal after 4 h reaction time (**Figure S1c**). In addition, the main reaction product is decane hydroperoxide, a reaction intermediate that was not observed in the presence of NO.

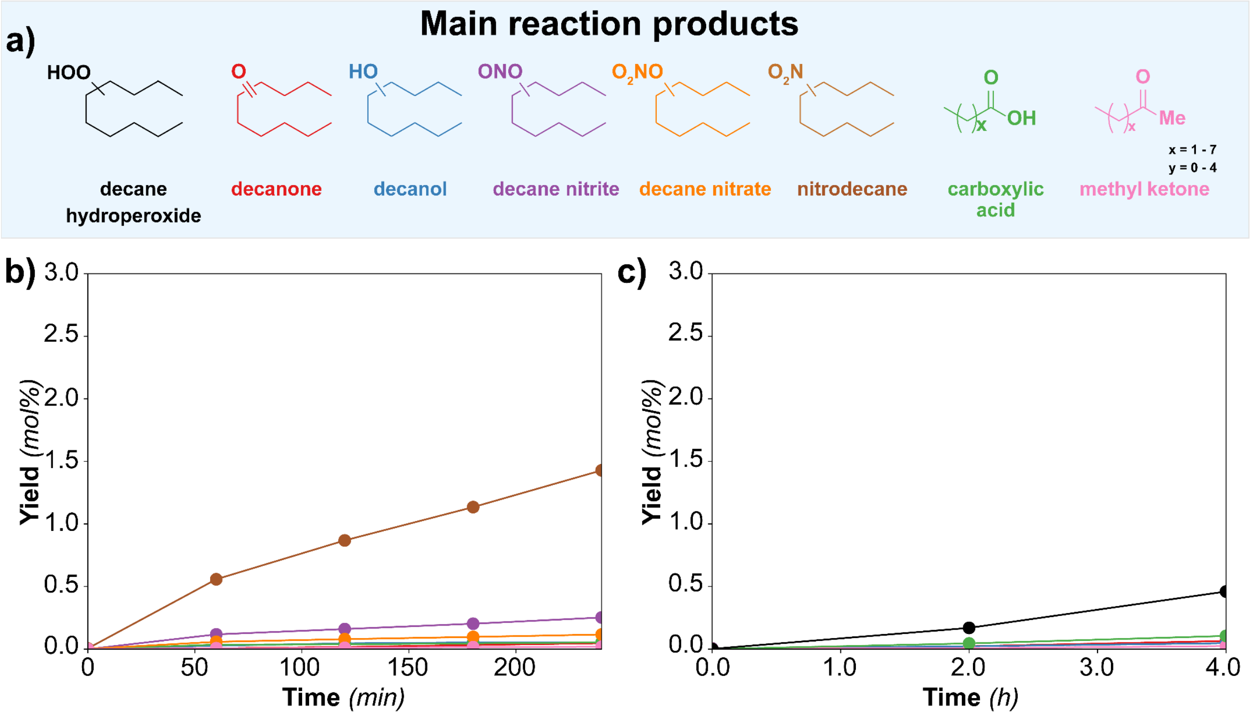

Figure S1. (a) Main reaction products observed in the decane oxidation experiments, with molecule colors corresponding to the line colors in the following plots. (b) Yield time course from gas chromatography-flame ionization detection (GC-MS/FID) data for decane oxidation with NO/N_2 only at 140 °C (100 psi, NO/N_2 = flow (20% NO in N_2) = 25 mL/min. (c) Yield time course from GC-MS/FID for decane oxidation with O_2 only at 140 °C (100 psi, O_2 flow = 15 mL/min).

1.2 Functional group distribution during decane oxidation

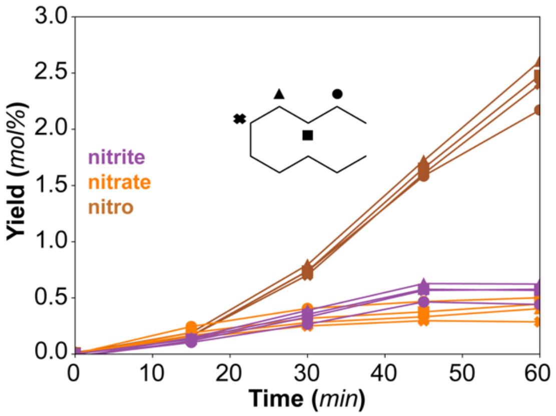

Figure S2. The functional group distribution over the different secondary positions in decane oxidized under standard conditions (*i.e.*, 140 °C, 100 psi, O_2 flow = 15 mL/min, NO flow (20% in N_2) = 25 mL/min.)

1.3 Analysis of C–C cleavage of ketone intermediates

2-Decanone-3-oxime is a potential product of 2-decanone oxidation through an electrophilic process similar to that of proposed for HNO_3-promoted cyclohexanone oxidation. 2-Decanone-3-oxime was synthesized, and it was then subjected to cyclohexane autoxidation conditions. Some of the 2-decanone-3-oxime is converted to 2,3-decanedione, while the majority (approximately 75%) undergoes C–C cleavage, leading to formation of either octanitrile or octanoic acid (**Figure S3a**). A control experiment showed that nitriles do not react further under standard conditions (**Figure S3b**) (*i.e.*, 140 °C, 100 psi, O_2 flow = 15 mL/min, NO/N_2 flow (20% NO in N_2) = 25 mL/min). These observations, together with the lack of significant nitrile formation during decane oxidation, suggest that α-keto oximes are not major reaction intermediates under the O_2/NO conditions. This conclusion further suggests that the radical reactivity outline in the main text of the manuscript is more likely than electrophilic reactivity en route to C–C cleavage.

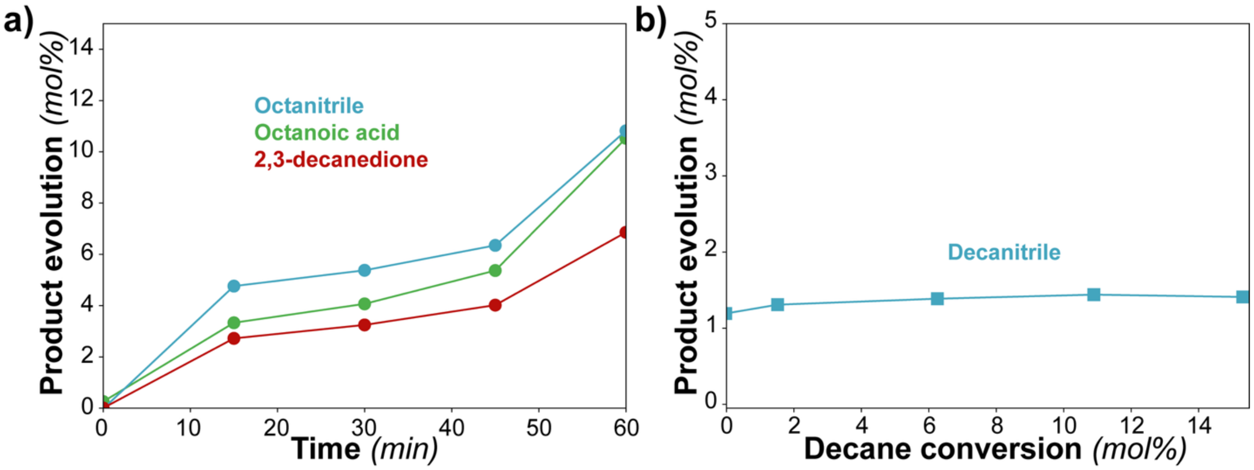

Figure S3. (a) Analysis of the reactivity of 1% 2-decanone-3-oxime at 100 °C in 10 mL cyclohexane (100 psi, O_2 flow = 15 mL/min, NO/N_2 flow (20% NO in N_2) = 25 mL/min). (b) Analysis of the reactivity of decanitrile (~100 mg in 10 mL decane) as a function of decane conversion under standard reaction conditions (140 °C, 100 psi, O_2 flow = 15 mL/min, NO/N_2 flow (20% NO in N_2) = 25 mL/min).

Oxidation of 6-undecanone under O_2/NO conditions produces exclusively C_6 and C_5 monocarboxylic acids in an approximately 1:1 ratio. This well-defined product distribution is consistent with selective cleavage of the C–C bond α to the ketone, rather than nonselective radical fragmentation pathways.

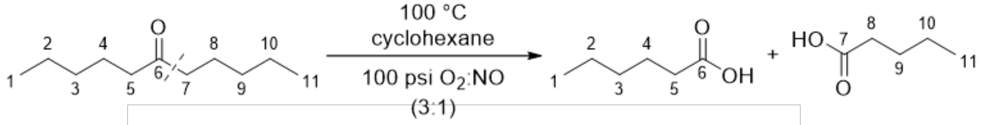

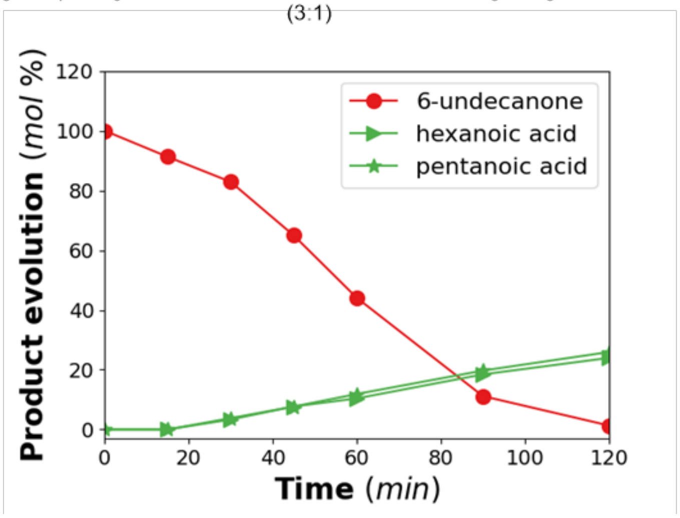

Figure S4. Analysis of the reactivity of 1% 6-undecanone at 100 °C in 10 mL cyclohexane (100 psi, O_2 flow = 15 mL/min, NO/N_2 flow (20% NO in N_2) = 25 mL/min). Partially oxidized intermediates are detected by GC–MS but are not quantified in this analysis; they are presumed to account for the missing mass balance.

To gain further insight into the importance of the α-position in ketone oxidation, a deuterium kinetic isotope study was performed. The oxidation rates of 6-undecanone and 6-undecanone-5,5,7,7-d4 were compared in separate oxidation experiments. To minimize experimental variability, the data were normalized to the rate of cyclohexyl nitrate formation, a major product arising from the background oxidation of the cyclohexane solvent. This normalization provides a more reliable proxy for overall oxidation progress. By normalizing the change in ketone concentration to the corresponding formation of cyclohexyl nitrate, the slopes of the resulting fits were used to determine the relative oxidation rates. A KIE of 3.2 was observed.

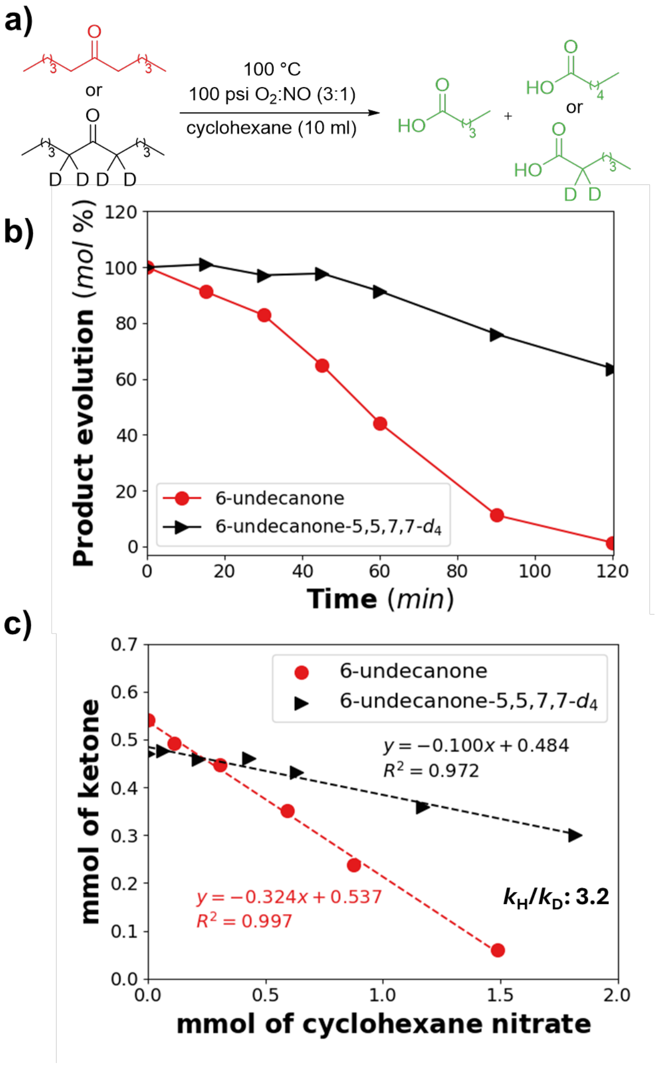

Figure S5. a) Reaction scheme for kinetic isotope experiment (KIE) on 6-undecanone. b) Analysis of the reactivity of 1% 6-undecanone or 6-undecanone-5,5,7,7-*d*4 at 100 °C in 10 mL cyclohexane (100 psi, O_2 flow = 15 mL/min, NO/N_2 flow (20% NO in N_2) = 25 mL/min). c) Relative rates of ketone and deuterated ketone consumption normalized to cyclohexane nitrate formation. Comparison of the non-deuterated and deuterated substrates yields a KIE of 3.2.

To gain further insight into the role of enolization in ketone oxidation, the rates of enolization of 6-undecanone and 6-undecanone-5,5,7,7-d4 were compared to obtain a rate of enolization and an enolization kinetic isotope effect (KIE). Because carboxylic acids are the major products of the reaction and are known to promote enolization, 20 vol% acetic acid was added to the reaction mixture to better mimic the oxidative reaction environment. The reaction was conducted under N_2 at 100 °C to suppress oxidation, using conditions similar to those in **Figure S5**, which showed efficient conversion of the ketone. Under these conditions, only minimal enolization was observed for both the non-deuterated and deuterated ketones. These results suggest that acetic acid alone does not facilitate enolization at a sufficiently rapid rate for this pathway to account for ketone oxidation. However, HNO_3 is also expected to be present under the oxidative reaction conditions, and its influence on enolization was therefore investigated in **Figure S7**.

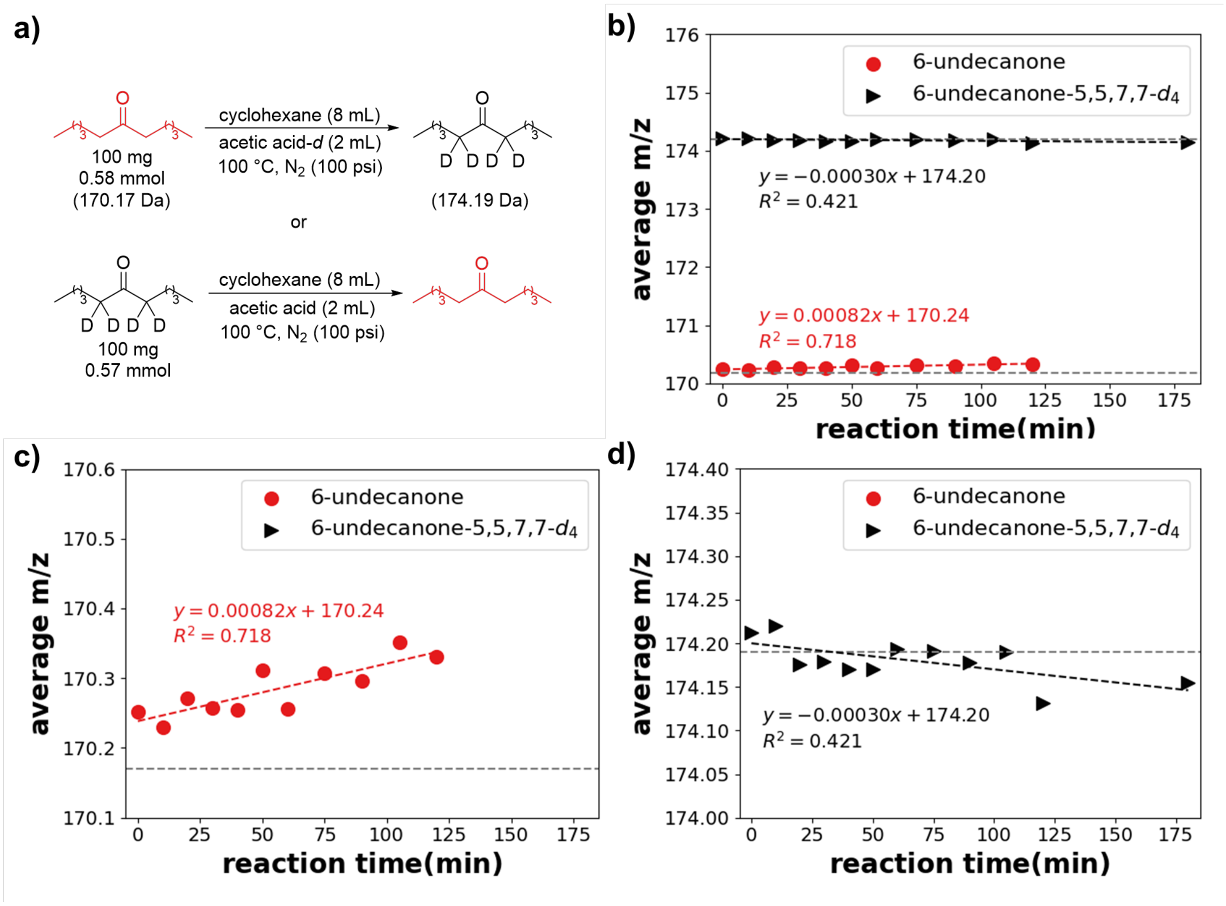

Figure S6. a) Reaction scheme for kinetic isotope experiment (KIE) for enolization. b) Analysis of the reactivity of 6-undecanone or 6-undecanone-5,5,7,7-d_4 at 100 °C in 8 mL cyclohexane and 2 mL acetic acid (100 psi N_2). c) Zoom-into the low molecular range of panel b. d) Zoom-into the high molecular range of panel b.

Prior experiments (**Figure S6**) examined the rate of enolization in the presence of 20 vol% acetic acid. However, under O_2/NO oxidation conditions, both H_2O and HNO_3 are generated and may additionally catalyze enolization. To better replicate the oxidative reaction environment, 6-undecanone and 6-undecanone-5,5,7,7-d_4 were heated at 100 °C under N_2 in a reaction mixture containing cyclohexane (8 mL), acetic acid (1.5 mL), H_2O (0.4 mL), and HNO_3 (0.1 mL, 70 wt% in H_2O). Reaction progress was monitored by GC–MS by tracking the average molecular weight distribution of the ketone isotopologues over the m/z range of 170–175 Da, corresponding to undeuterated 6-undecanone (170.17 Da) and deuterated species derived from 6-undecanone-5,5,7,7-d_4 (174.19 Da). Under these conditions, significant enolization was observed on a timescale comparable to ketone oxidation (**Figure S5**). The reaction exhibited apparent zero-order kinetics and gave a kinetic isotope effect (KIE) of 4.9.

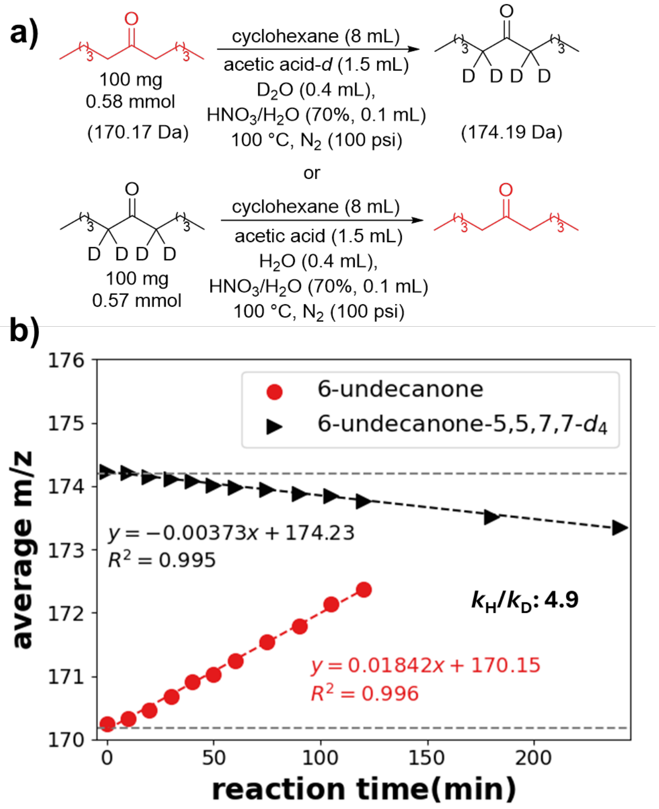

Figure S7. a) Reaction scheme for kinetic isotope experiment (KIE) for enolization. b) Analysis of the reactivity of 6-undecanone or 6-undecanone-5,5,7,7-d_4 at 100 °C in 8 mL cyclohexane, 1.5 mL acetic acid, 0.4 mL H_2O and 0.1 ml HNO_3 (100 psi N_2). c) Relative rates of ketone and deuterated ketone consumption normalized to cyclohexane nitrate formation. Comparison of the non-deuterated and deuterated substrates yields a KIE of 3.2.

2. GENERAL EXPERIMENTAL

2.1 Materials and Reagents

All reagents were purchased and used as received unless otherwise noted.

Chemical	Supplier	Purity
Decane	Thermo scientific	99%
Cyclohexane	Spectrum chemicals mfg corp	ACS grade
3-decanol	Thermo scientific	97%
4-decanol	Tokyo Chemical Industry	>98%
3-decanone	Tokyo Chemical Industry	>98%
Decanoic acid	Sigma Aldrich	>98%
Nonanoic acid	Sigma Aldrich	>97%
Octanoic acid	Sigma Aldrich	>99%
Decanitrile	Tokyo Chemical Industry	>98%
Hydroxylamine hydrochloride	Sigma Aldrich	99%
Sodium nitrite	Chem Impex	99,4%
Sulfuric acid	Sigma Aldrich	95 – 98%
Nitric acid	Acros Organics	70% in H_2O
Hydrochloric acid	Sigma Aldrich	37% in H_2O
Hydrobromic acid	Sigma Aldrich	48% in H_2O
Potassium hydroxide	Sigma Aldrich	>85%
K_2CO_3	Sigma Aldrich	>99%
Na_2SO_4	Sigma Aldrich	>97%
Dichloromethane (DCM)	Fisher Chemicals	ACS grade
Ethyl acetate	Fisher Chemicals	ACS grade
Hexanes	Fisher Chemicals	ACS grade
Pentane	Fisher Chemicals	ACS grade
Acetonitrile	Sigma Aldrich	HPLC grade
Ethanol	Oakwood chemicals	ACS grade
Tetrahydrofuran (THF)	Sigma Aldrich	HPLC grade
Dimethyl sulfoxide (DMSO)	Sigma Aldrich	HPLC grade
NO/N_2 (20/80)	Airgas Midwest	Industrial grade

O_2	Airgas Midwest	Industrial grade
N-methyl-N-trimethylsilyltrifluoroacetimide	Tokyo Chemical Industry	>95%
PPh_3	Sigma Aldrich	99%
CCl_3D	Sigma-Aldrich	99.8%
High-density polyethylene	Sabic	M_n = 16010 g/mol M_w = 137500 g/mol
Methanol	Sigma Aldrich	>99%
Acetyl chloride	Sigma Aldrich	>99%
4-heptanone	Sigma Aldrich	>98%

2.2 Equipment and Instrumentation

Nuclear magnetic resonance experiments

NMR measurements were performed on a Bruker Avance III 500 MHz spectrometer. Resonances were referenced to residual solvent peaks (^{1}H: δ 7.26 ppm, $^{13}C\{1H\}$: δ 77.16 ppm for $CDCl_3$). Chemical shifts (δ) are given in ppm and coupling constants (J) are quoted in hertz (Hz). Resonances are described as s (singlet), d (doublet), t (triplet), q (quartet), br (broad singlet) and m (multiplet) or combinations thereof.

Gas chromatography-mass spectrometry/gas chromatography flame ionization detection

Gas chromatography experiments on decane samples were performed using an Agilent 8890 GC system coupled to 5977C mass spectrometer (MS)/flame ionization detector (FID) and fitted with an DB-35ms Ultra Inert column (30 m, 0.320 mm, 0.25 µm; Part No. 123-3832UI). Helium gas was used as a carrier gas at a flow rate of 2.0 mL/min. Samples were injected in at an injector temperature of 200 °C. The oven temperature program profile was as follows: column oven was kept at 40 °C for 4 min (initial temperature), increased at 2 °C/min to 125 °C and increased at 40 °C/min to 300 and was hold for 3 min.

Qualitative GC-MS analysis was performed with Agilent 8890 GC system equipped with MS 5977C (electron ionization (EI) mode) to identify the products. After identifying the products, quantitative analysis was performed with flame ionization detectors (FID).

Gas chromatography (GC) on oxidized polyethylene samples were performed on a GC x GC (Shimadzu, GC-2010 Plus) coupled with an FID and MS (Shimadzu, GCMS-QP2010 Ultra). Samples were injected at an injector temperature of 200 °C. The products were separated using a GC 60m, 0,25 mm ID, 0.25 µm column and a VF-5ms, 30 m, 0.25 ID, 0.25 µm column. The column oven temperature was kept at 40 °C for 5 min, increased at 10 °C/min to 280 °C and hold for 10 min.

On both GC instruments the qualitative analysis was performed with the FID signals by using effective carbon number (ECN) theory. With this theory response factors can be calculated in cases where pure product mixtures are not readily available for detector calibration.[1,2] As a consequence of the FID mechanism, each individual carbon atom will give a linear response depending on its chemical nature, which makes that response factors can be calculated with excellent accuracy.[3] Literature references of alkyl nitrite and alkyl nitrate were not found, therefore the same value as for secondary alcohols was taken,

namely -0.75. The yields were calculated the response factors obtained from ECN theory and an internal standard

Infrared spectroscopy

IR spectra of crude decane reaction mixture were recorded on a Bruker Tensor 27 in ATR-mode. The polyethylene samples were measured in ATR mode on a Perkin Elmer FT-IR Frontier spectrometer with MCT detector, while using a Perkin Elmer Universal ATR sampling accessory.

Elemental analysis (CHN)

The crude reaction mixtures were submitted to MEDAC Ltd to execute the measurements. The results were used as received and the carbon recovery was calculated by multiplying the percentage of carbon in the sample times the sample weight.

2.3 Safety Considerations

Nitric oxide gas

Nitric oxide is a highly toxic and potentially explosive colorless gas. To minimize explosion risk, it is used in a diluted nitrogen stream. NO rapidly reacts with O_2 to form nitrogen dioxide (NO_2), a reddish-brown gas that remains difficult to detect and is also highly toxic. This gas should only be used in small quantities and in well-ventilated areas capable of handling both normal operation and potential system failures.

Additionally, NO/NO_2 gas is incompatible with several materials and can degrade PEEK tubing (used in some cases in this study) with prolonged exposure. Fittings and tubing should be monitored regularly for signs of degradation.

Pressurized O_2 gas

Several oxidation conditions in this study (e.g., 100 psi O_2) exceed the experimental limiting oxygen concentration for aerobic oxidation (Org. Process Res. Dev. 2015, 19, 1537), significantly increasing the risk of highly energetic ignitions or even explosions. These limits were exceeded in our small-scale experiments, with safety precautions including the use of blast shields.

Peroxides

The reactions described in this paper involve peroxides, which can be explosive when concentrated. Extreme caution should be exercised when handling concentrated samples, and working with dilute solutions is preferred. Peroxides should be properly quenched with appropriate reagents (e.g., PPh_3, PMe_3, or $Na_2S_2O_3$) before disposal.

3. SYNTHESIS OF MODEL COMPOUNDS

3.1 Synthesis of 3-decanone oxime

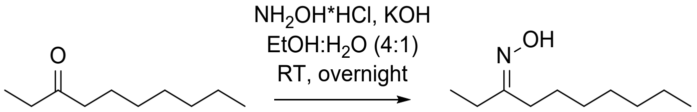

781 mg decanone (5 mmol, 1 equiv.), 695 mg $NH_2OH \cdot HCl$ (10 mmol, 2 equiv.), 842 mg KOH (15 mmol, 3 equiv.) and 50 mL ethanol/H_2O (4:1) were added to a round bottom flask. The colorless suspension was stirred overnight. Then, the ethanol was evacuated and the mixture was extracted two times with 10 mL DCM. The combined DCM fraction was dried with Na_2SO_4, filtered and the solvent was removed under vacuum. The product was obtained as a colorless oil in 99% yield (847 mg). The characterization of the oxime group matches literature values for linear alkyl oximes.[4]

^1H NMR (500 MHz, $CDCl_3$) δ 9.67 (d, J = 25.5 Hz, 1H), 2.40 – 2.29 (m, 2H), 2.26 – 2.11 (m, 2H), 1.49 (m, 2H), 1.40 – 1.18 (m, 8H), 1.11 – 1.02 (m, 3H), 0.91 – 0.82 (m, 3H).

^{13}C NMR (126 MHz, $CDCl_3$) δ 162.77 (dd, J = 19.8, 2.8 Hz), 33.82, 31.88, 29.99, 29.42, 29.20, 29.17, 27.73, 27.55, 26.38, 25.77, 22.76, 20.92, 14.19, 10.84, 10.21.

ATR IR: v = 3246 (m), 2923 (s), 2855 (s), 1657 (w), 1459 (s), 1377 (m), 1340 (m), 1113 (m), 939 (s), 723 (s) cm^{-1}.

*Both E and Z isomers of the oxime were observed

3.2 Synthesis of 2-nitro decane

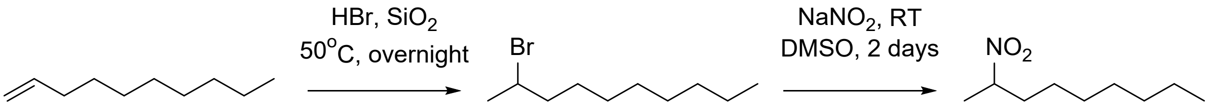

The procedure was adapted from the following references.[5,6] 15 g silica were added to a vial, which was cooled to 0 °C with an ice bad. Subsequently, 842 mg 1-decene (6 mmol, 842 mg) were added dropwise while shaking. Then, over a period of 15 min 2.7 mL 48 % HBr (24 mmol, 4.04 g, 4 equiv.) were added dropwise while shaking. In order for the reaction to go to completion the vial was heated to 50 °C for 1 day. The next day, the SiO_2 was extracted three times with 50 mL hexane and the solvent was removed under reduced pressure. The colorless oil was redissolved in 5 mL DMSO and 840 mg $NaNO_2$ (12 mmol, 2 equiv.) were added. After stirring for 2 days, the solution was added to 10 mL H_2O and the mixture was extracted twice with 10 mL hexane. The crude product mixture was purified by column chromatography (100 % hexanes). The product was obtained as a colorless oil in a combined yield over two steps of 31 % (320 mg, 1.85 mmol). The final product used for the spiking experiments contained ~8 % 1-nitrodecane and for full characterization of 2-nitro decane the compound was purified by prep TLC (DCM/hexanes:30/70). The characterization of nitro group matches literature values for secondary nitro alkyl.[6]

^1H NMR (500 MHz, $CDCl_3$) δ 4.56 (m, 1H), 1.99 (m, 1H), 1.70 (m, 1H), 1.52 (d, J = 6.6 Hz, 3H), 1.36 – 1.18 (m, 13H), 0.87 (t, J = 6.9 Hz, 3H).

^{13}C NMR (126 MHz, $CDCl_3$) δ 83.75, 35.33, 31.92, 29.39, 29.26, 29.12, 25.82, 22.77, 19.37, 14.25.

ATR IR: v = 2957 (m), 2927 (s), 2857 (m), 1551 (s), 1465 (w), 1390 (w), 1359 (w) cm^{-1}.

3.3 Synthesis of decane-3-nitrate

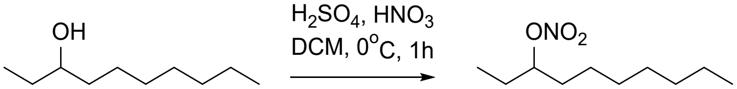

The procedure was adapted from the following reference.[7] 792 mg 3-decanol (5 mmol, 1 equiv.) were dissolved to in 30 mL DCM and cooled to 0 °C with an ice bad. Subsequently, 5 mL 98 % H_2SO_4 were added and the colorless solution was stirred for 5 min. Then 1 mL 70 % HNO_3 in 10 mL DCM was added dropwise and the mixture was stirred for 1 h. The slightly yellow mixture was added to a separatory funnel and the mixture was washed with three times with 20 mL H_2O. The organic fraction was filtered over silica, dried with Na_2SO_4, filtered and the solvent was evacuated under reduced pressure. The product was obtained as a colorless to pale yellow oil in 44 % yield (448 mg, 2.20 mmol). The characterization data matches a literature refence.[8]

^{1}H NMR (500 MHz, $CDCl_3$) δ 4.96 (ddd, J = 12.3, 6.7, 5.6 Hz, 1H), 1.77 – 1.54 (m, 4H), 1.45 – 1.16 (m, 10H), 0.96 (t, J = 7.5 Hz, 3H), 0.91 – 0.83 (m, 3H).

^{13}C NMR (126 MHz, $CDCl_3$) δ 86.32, 31.93, 31.85, 29.49, 29.23, 25.51, 25.19, 22.76, 14.22, 9.46.

ATR IR: v = 2985 (w), 2957 (m), 2926 (m), 2855 (m), 1621 (s), 1554 (w), 1465 (w), 1381 (w), 1354 (w), 1274 (s), 1119 (w), 860 (s), 758 (w), 700 (w) cm^{-1}.

3.4 Synthesis of 3-decanyl nitrite

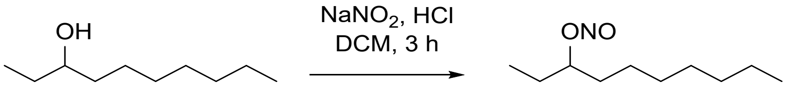

792 mg 3-decanol (5 mmol, 1 equiv.), $NaNO_2$ (380 mg, 5.5 mmol, 1.1 equiv.), H_2O (1080 mg, 60 mmol, 12 equiv.) were added to a round bottom flask. Subsequently, the mixture was cooled to 0 °C followed by the dropwise addition of HCl (2.1 mL, 25 mmol, 5 equiv.). After 3h, the yellow suspension was added dropwise to a 10 mL K_2CO_3 solution and neutralized. The product was extracted with 10 mL DCM. Then, the organic fraction dried with Na_2SO_4, filtered and DCM was removed under vacuum. Thereafter, the product was redissolved in pentane, filtered over silica and the pentane was evacuated under reduced pressure. The product was obtained as a colorless to pale yellow oil in 65 % yield (610 mg, 3.26 mmol). The characterization of nitrite group matches literature values for secondary alkyl nitrite.[9]

^{1}H NMR (500 MHz, $CDCl_3$) δ 5.38 – 5.26 (m, 1H), 1.84 – 1.60 (m, 4H), 1.42 – 1.15 (m, 10H), 0.99 – 0.78 (m, 6H).

^{13}C NMR (126 MHz, $CDCl_3$) δ 81.52, 33.62, 31.27, 28.78, 27.08, 24.89, 22.15, 21.87, 13.59, 9.33.

ATR IR: v = 2959 (w), 2927 (w), 2856 (w), 1716 (w), 1637 (m), 1461 (w), 1350 (w), 1276 (w), 911 (w), 866 (w), 770 (s), 724 (m), 682 (w), 635 (m) cm^{-1}.

3.5 Synthesis of 2-decanone-3-oxime

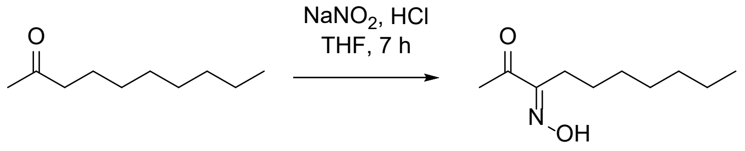

469 mg 2-decanone (3 mmol, 1 equiv.), 207 mg $NaNO_2$ (3 mmol, 1 equiv.) and 6 mL THF were added to a vial. Subsequently, the vial was cooled to 0 °C with an ice bath and dropwise 3.9 mL 37% HCl were added. After 2 h the mixture was allowed to go to room temperature and the mixture was stirred for another 5 h. Then, the mixture was added to 15 mL H_2O and extracted twice with 15 mL ethyl acetate. The combined organic layers were washed with a $NaCO_3$ solution and the solvent was removed under reduced pressure. The product was purified by column chromatography (Hexanes:EtOAc/9:1). The product was obtained as a colorless solid in 51 % yield (281 mg, 1.52 mmol). The characterization data matches a literature refence.[10]

^{1}H NMR (500 MHz, $CDCl_3$) δ 8.84 (s, 1H), 2.58 – 2.50 (m, 2H), 2.36 (s, 3H), 1.47 – 1.37 (m, 2H), 1.32 – 1.17 (m, 8H), 0.86 (t, J = 7.1 Hz, 3H).

^{13}C NMR (126 MHz, $CDCl_3$) δ 197.25, 160.85, 31.85, 29.91, 29.12, 26.04, 25.53, 22.76, 22.67, 14.23.

ATR IR: v = 3257 (w), 3174 (m), 3043 (m), 2950 (m), 2913 (m), 2849 (m), 1661 (s), 1457 (m), 1374 (m), 1335 (w), 1272 (w), 1234 (w), 1141 (m), 1075 (m), 990 (s), 953 (m), 813 (m), 722 (w) cm^{-1}.

3.6 Synthesis of decane-3-hydroperoxide

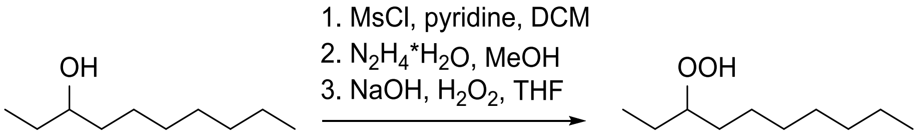

The following reference was used for the synthesis of decane-3-hydroperoxide.[11] 1900 mg 3-decanol (12 mmol, 1 equiv.) and 1898 mg pyridine (24 mmol, 2 equiv.) were dissolved in 25 mL DCM and cooled to 0 °C with an ice bad. Subsequently, dropwise 1650 mg methane sulfonyl chloride (14.4 mmol, 1.2 equiv.) were added and the reaction mixture was stirred for one hour yielding a colorless solution. Then, the reaction mixture was quenched with 24 mL 10 % HCl and the mixture was extracted with ether and washed with a $NaHCO_3$ solution and H_2O. The organic fraction was dried with Na_2SO_4 and the solvent was evaporated under reduced pressure.

Then, 10 mL methanol and 10.32 g N_2H_4*H_2O were added to a round bottom flask. The flask was sealed and heated to 90 °C for 1.5 h. Subsequently, the reaction mixture was extracted with ether and washed with 50% KOH and H_2O and the solvent was removed under reduced pressure.

In the third step, the crude product was redissolved in 140 mL THF, 1g KOH and 220 mL 30 wt.% aq H_2O_2. The reaction mixture was stirred for 3 days. Then the product was extracted with DCM, washed with H_2O, dried with Na_2SO_4 and the solvent was evaporated under reduced pressure. The product was further purified by column chromatography and was obtained as a colorless oil in 49 % yield. The characterization data matches a literature refence.[11]

^{1}H NMR (500 MHz, CDCl3) δ 7.56 (m, 1H), 3.84 (p, J = 5.5 Hz, 1H), 1.71 – 1.52 (m, 3H), 1.52 – 1.43 (m, 1H), 1.41 – 1.20 (m, 10H), 0.92 (t, J = 7.5 Hz, 3H), 0.87 (t, J = 7.0 Hz, 3H).

^{13}C NMR (126 MHz, $CDCl_3$) δ 86.98, 31.97, 31.53, 29.86, 29.39, 25.57, 24.81, 22.80, 14.26, 9.63.

3.7 Synthesis of 5,6-decane dione

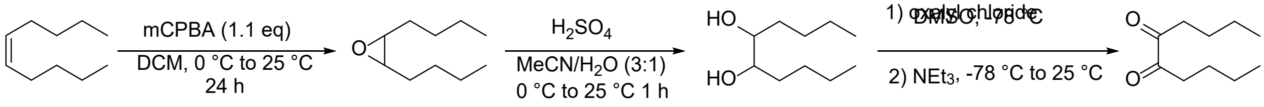

mCPBA epoxidation and ring opening

The following literature procedures were used for the synthesis of 5,6-decane dione.[12,13] m-CPBA (4.43 g, 25.7 mmol, 1.2 equiv.) and DCM (70 ml) were placed in a round-bottom flask. The reaction mixture was cooled to 0 °C and 5-decene (3.0 g, 21.41 mmol, 1 eq) was slowly added (15 min). The reaction was allowed to stir at 0 °C for 1 h before allowing it to warm to 25 °C. After 12 h the reaction mixture was cooled back to 0 °C and saturated $Na_2S_2O_3$ aqueous solution was added. The crude mixture was filtered and washed with saturated $NaHCO_3$ aqueous solution three times. After washing with brine, it was dried with Na_2SO_4, the organic phase was concentrated under reduced pressure. This crude epoxide was used directly for the next hydrolysis reaction.

To the round-bottom flask containing an epoxide, MeCN/H_2O (v/v = 3:1, 100 ml) was added and cooled to 5 °C. Conc. H_2SO_4 aqueous solution (3.6 M, 5.95 ml, 21.41 mmol, 1.0 equiv.) was slowly added over 15 min. The reaction mixture was allowed to warm to 25 °C and stir for an additional 1 h. The reaction mixture was partially concentrated under reduced pressure (70% vol removed). The concentrated mixture was extracted by Et_2O three times. It was washed with brine three times and dried with Na_2SO_4. Concentration of the mixture under reduced pressure yielded, 2.61g (15 mmol, 70% yield) of 5,6-decandiol (contained trace m-chlorobenzoic acid).

Swern oxidation

Dimethyl sulfoxide (4.68 g, 4.3 ml, 60 mmol, 4 eq) was added to a stirred solution of oxalyl chloride (5.71 g, 3.9 ml, 45 mmol, 3 eq) in dichloromethane (240 ml) at -78°C. After 5 min, a solution of 5,6-decandiol (2.61 g, 15 mmol, 1 eq) in dichloromethane (80 ml) was slowly added (over period of 60 min) and the resulting solution was stirred at -78°C for 1 h. Then triethylamine (9.11 g, 12.6 ml, 90 mmol, 6 eq) was added, the mixture was stirred for 1 h at -78°C and then warmed to ambient temperature. The reaction mixture was washed with water, organic layer was dried over Na_2SO_4, filtered and evaporated. The product was purified by column chromatography using a gradient from pure cyclohexane to pure DCM to obtain 0.95 g (5.58 mmol, 37 % yield) of 5,6-decadione. The characterization data matches a literature reference.[14]

^{1}H NMR (500 MHz, $CDCl_3$) δ 2.73 (t, J = 7.4 Hz, 1H), 1.61 – 1.50 (m, 1H), 1.39 – 1.27 (m, 1H), 0.91 (t, J = 7.4 Hz, 2H).

^{13}C NMR (126 MHz, $CDCl_3$) δ 200.30, 35.94, 25.23, 22.40, 13.97.

ATR IR: *ν* = 2960 (m), 2873 (w), 1710 (s), 1465 (w), 1401 (m), 1117 (w), 1003 (w), 914 (w).

3.8 Synthesis of 6-undecanone-5,5,7,7-*d₄*

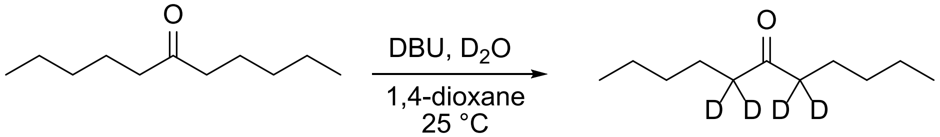

The following literature procedures were used for the synthesis of 6-undecanone-5,5,7,7-d_4.[14] 6-undecanone (0.5 g, 2.9 mmol, 1 eq.) was dissolved in a mixture of 6 mL of 1,4-dioxiane and D_2O (3.6 g, 200 mmol, 70

eq). DBU (1,8-diazabicyclo[5.4.0]undec-7-ene; 88 mg, 0.58 mmol, 0.20 equiv) was then added, and the reaction mixture was heated to 50 °C and stirred for 24 h. Upon completion, the reaction mixture was extracted with dichloromethane, and the combined organic layers were dried over $MgSO_4$, filtered, and concentrated under reduced pressure. The crude product was purified by passing through a silica plug, eluting with 2% ethyl acetate in hexanes. Removal of solvent afforded 6-undecanone-5,5,7,7-d_4 (460 mg; 2.6 mmol, 96% deuteration of alpha hydrogens to the ketone determined by loss of signal at 2.38 ppm).

4. OXIDATION EXPERIMENTS

4.1 Decane oxidation experiments

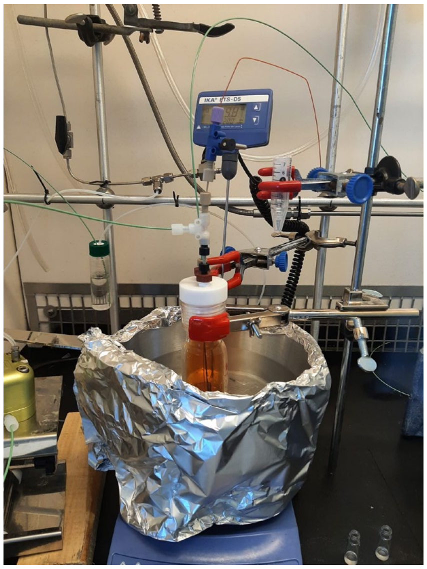

Oxidation experiments with decane were conducted in glass reactor vessels (Synthware™ round-bottom pressure vessel with a protective coating, 75 mL capacity, O.D. × L: 46 mm × 90 mm, bushing type #25, Synthware P170002D) equipped with a PTFE stir bar. Polyetheretherketone (PEEK) and/or Teflon tubing was used for gas delivery.

In a typical experiment, 10 mL of decane was loaded into the glass vessel. The reactor was first pressurized to 100 psi with O_2 using a backpressure regulator (Zaiput Flow Technologies), and the flow was set to 15 mL/min using a mass flow controller. The vessel was then lowered into an oil bath, stirring was initiated at 400 rpm, and NO (20% in N_2) was introduced at 25 ml/min. The pressure was maintained at 100 psi throughout the experiment.

The reaction time was recorded from the moment the first orange vapors entered the reaction vessel, with the system requiring approximately 15 min to reach a stable color. Throughout the experiment, multiple time points were collected by withdrawing ~0.3 mL samples from the vessel.

Spiking experiments with model substrates followed the same procedure, with ~1% of the target compound added. For more reactive model substrates, decane was replaced with cyclohexane to eliminate background signals from decane oxidation in GC analyses.

4.2 Decane GC-MS analysis

Samples for GC analysis of the products arising from decane oxidation were prepared by mixing 100 µL from the reactor with 100 µL ethyl acetate (EA) with the internal standard 1,2,3-trichlorobenzene (TCB) (50g TBE in 500ml of EA). For experiments at higher conversion 10 or 20 µL of this mixture was transferred to a new vial followed by the addition of 100 µL N-methyl-N-trimethylsilyltrifluoroacetimide. For the experiments with model substrates, 100 µL sample, 100 µL ethyl acetate with the internal standard 1,2,3-trichlorobenzene (TCB) and 100 µL N-methyl-N-trimethylsilyltrifluoroacetimide were mixed directly. Subsequently, the samples were heated at 60 °C for 1 h, after which the vials were filled up with ethyl acetate.

For the experiments performed with decane under neat conditions and decane hydroperoxide a second sample was prepared to determine hydroperoxide concentration. This sample was prepared in a similar way, but prior to the addition of methyl-N-trimethylsilyltrifluoroacetimide 100 µL PPh_3 in ethyl acetate (0.5 g in 2.5 mL) was added and allowed to react for 1 h at room temperature to reduce the peroxides to their corresponding alcohols. The peroxide concentration was determined by taking the difference in alcohol concentration between the two samples.

4.3 Polyethylene oxidation experiments

Oxidations with PE were performed in a 50 mL stainless steel (T316) Parr autoclave. In each experiment a glass liner was filled with approximately 200 mg HDPE and placed in the reactor. Then, the reactor was purged with N_2 and the reactor was pressurized with 20 bar NO/N_2 (20/80) followed by 20 bar O_2/NO (40/60). Subsequently, the reactor was heated to 100 °C in the first 20 min and to 120 °C in the next 40 min. Then, the reactor was kept for the desired time at 120 °C.

4.4 Polyethylene product analysis

From the crude product mixture obtained after PE oxidation approximately 30 % was kept separately for additional product analysis. The remaining of the crude reaction mixture was dissolved in 4 mL methanol, followed by the addition of 0.2 mL acetyl chloride and it was stirred for 1 h at 50 °C. When the conversion is sufficient the entire product mixture dissolves and clear solution was formed. Only at low conversions PE like solids were observed. After 1 h, one drop of the internal standard 4-heptanone was added. Prior to GC injection the sample was filtered in order to prevent the GC from clogging. The injection temperature of the GC was set to 200 °C. The yields are defined in the percentage of carbon atoms present in the PE starting material that ends up in the di-carboxylic acid product (equation 1).

$$Yield\ (mol\%) = \frac{C_{di-acids}}{C_{PE}} * 100\% \qquad (1)$$

The amount of recovered carbon in the oil fraction was calculated by multiplying the percentage of carbon obtained with CHN analysis times the sample weight of the crude reaction mixture (equation 2).

$$Carbon\ recovery\ (\%) = \frac{C_{oil\ fraction}}{C_{PE\ start\ material}} * 100\% = \frac{Weight\ oil\ fraction * \%C}{C_{PE\ start\ material}} * 100\% \qquad (2)$$

5. NMR, IR, AND MS SPECTRA

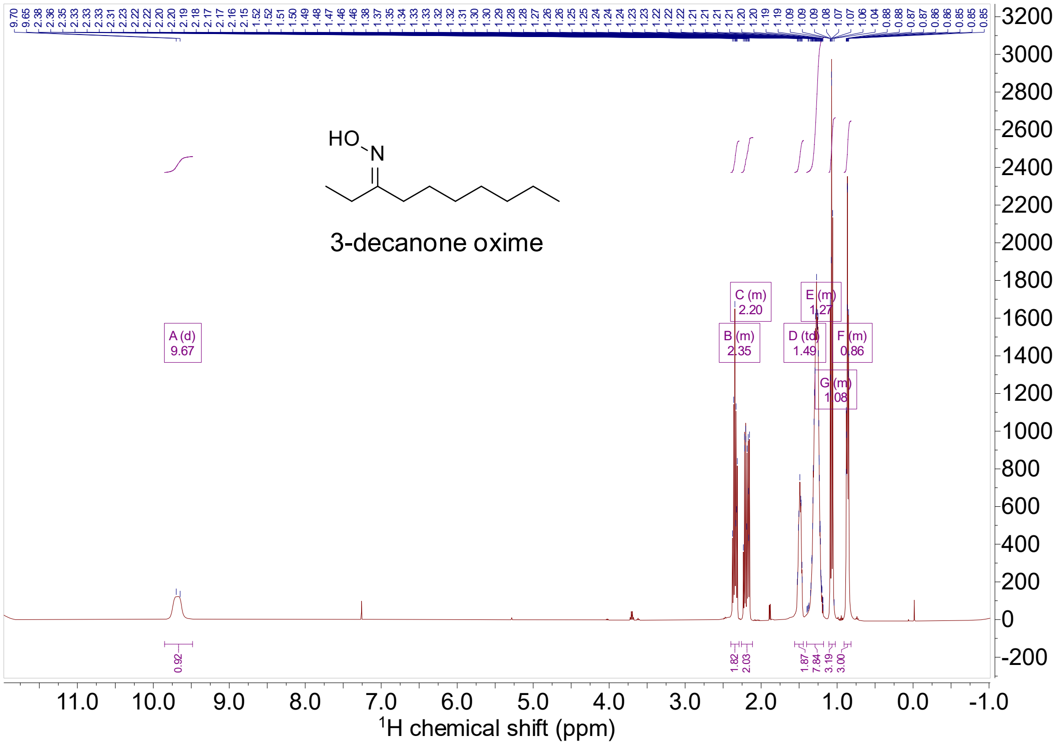

Figure S8. ^{1}H NMR spectrum of 3-decanone oxime.

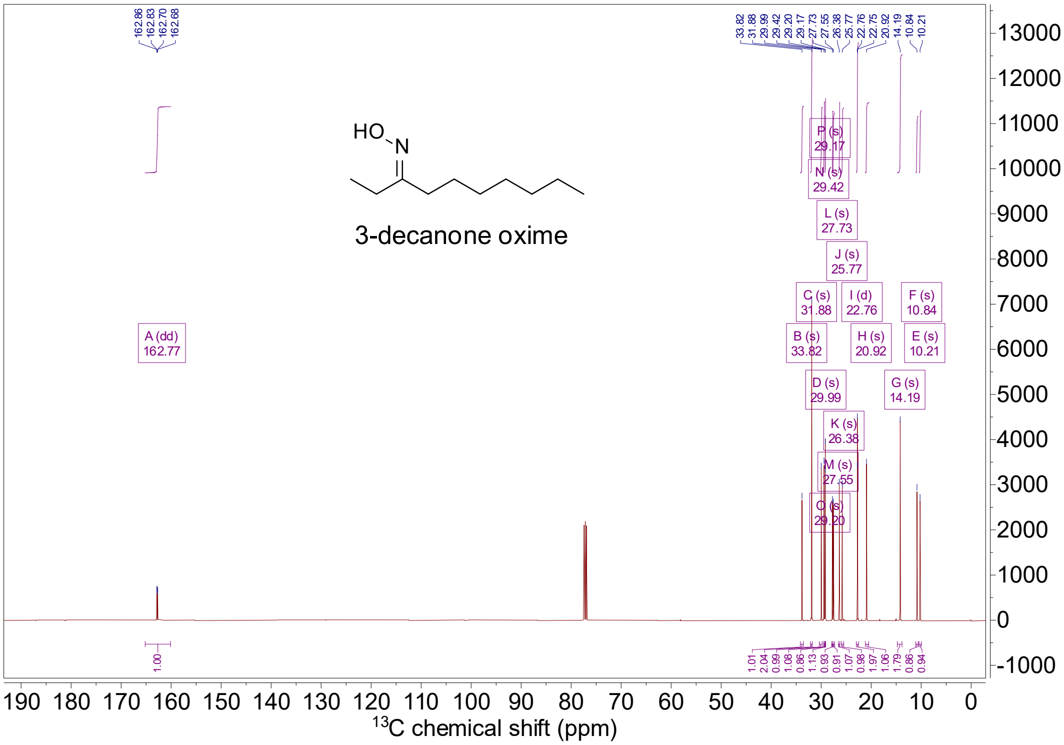

Figure S9. ^{13}C NMR spectrum of 3-decanone oxime.

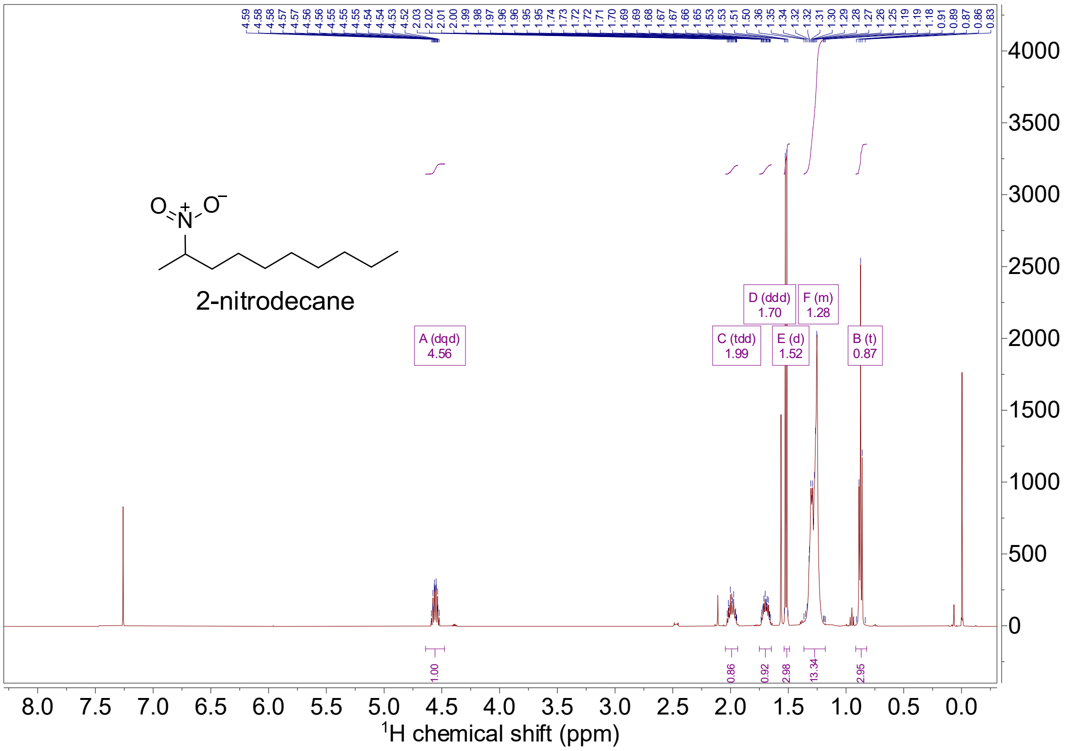

Figure S10. ^{1}H NMR spectrum of 2-nitrodecane.

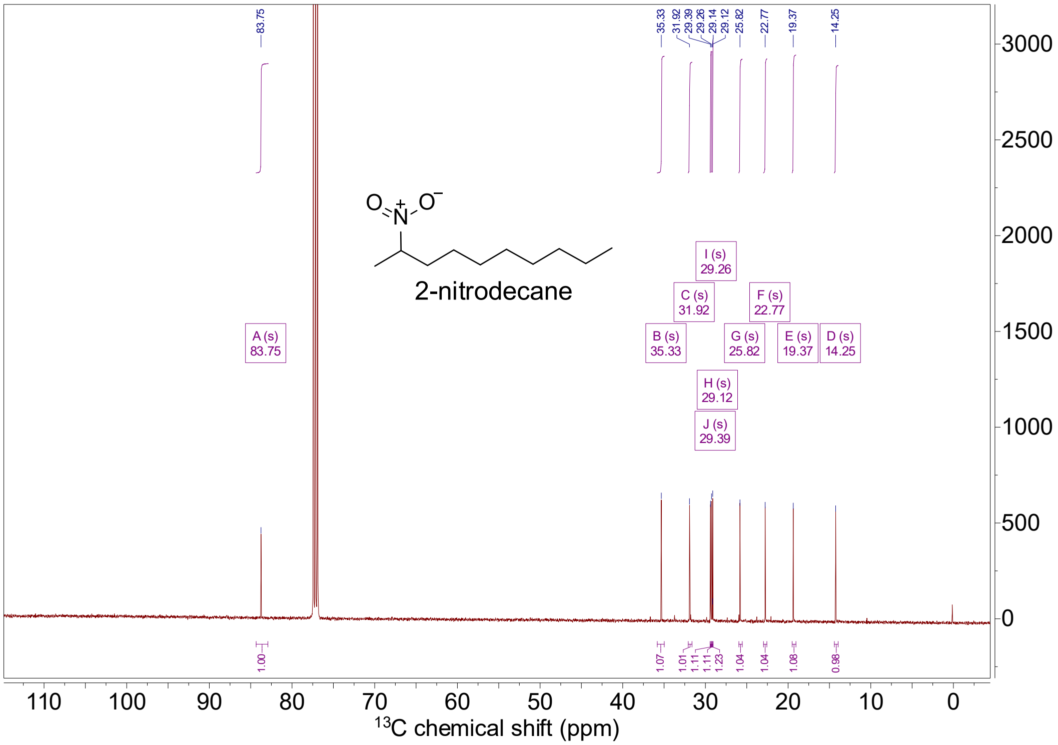

Figure S11. ^{13}C NMR spectrum of 2-nitrodecane.

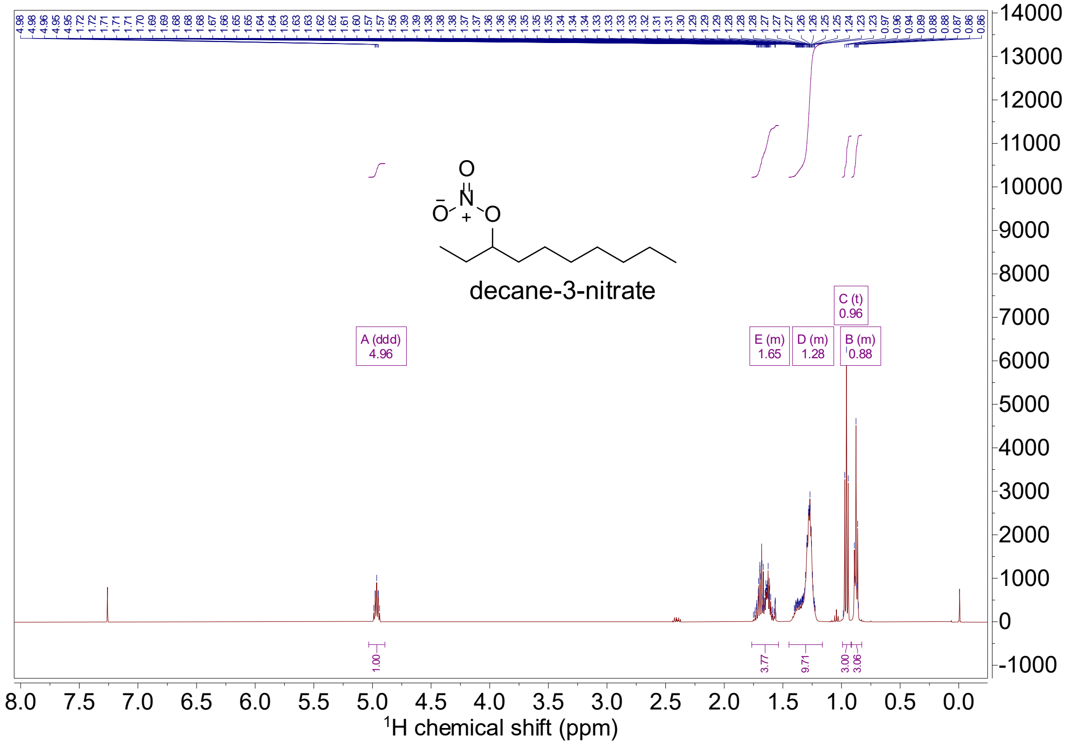

Figure S12. ^{1}H NMR spectrum of 3-decane nitrate.

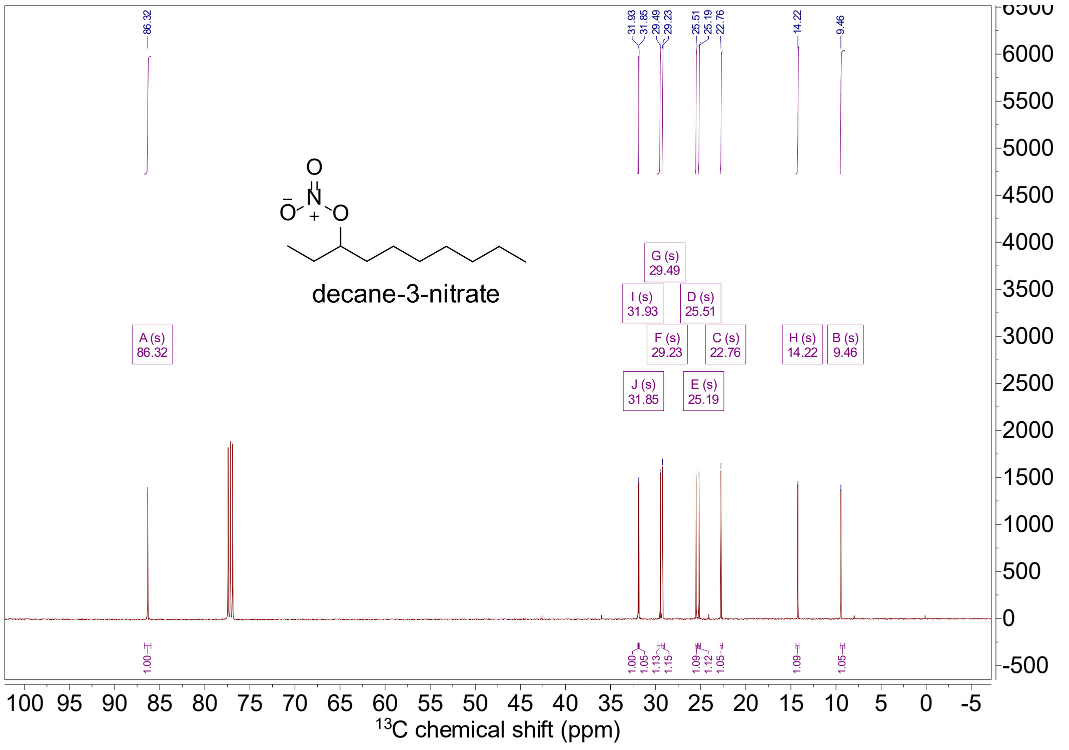

Figure S13. ^{13}C NMR spectrum of 3-decane nitrate.

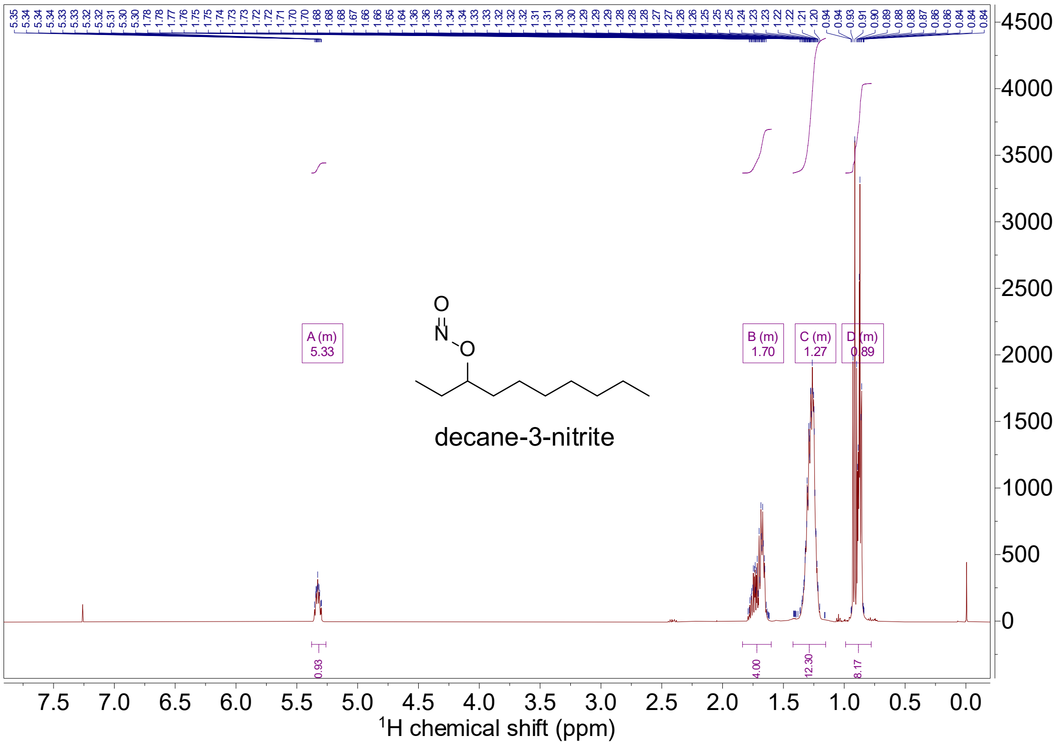

Figure S14. ^{1}H NMR spectrum of 3-decane nitrite.

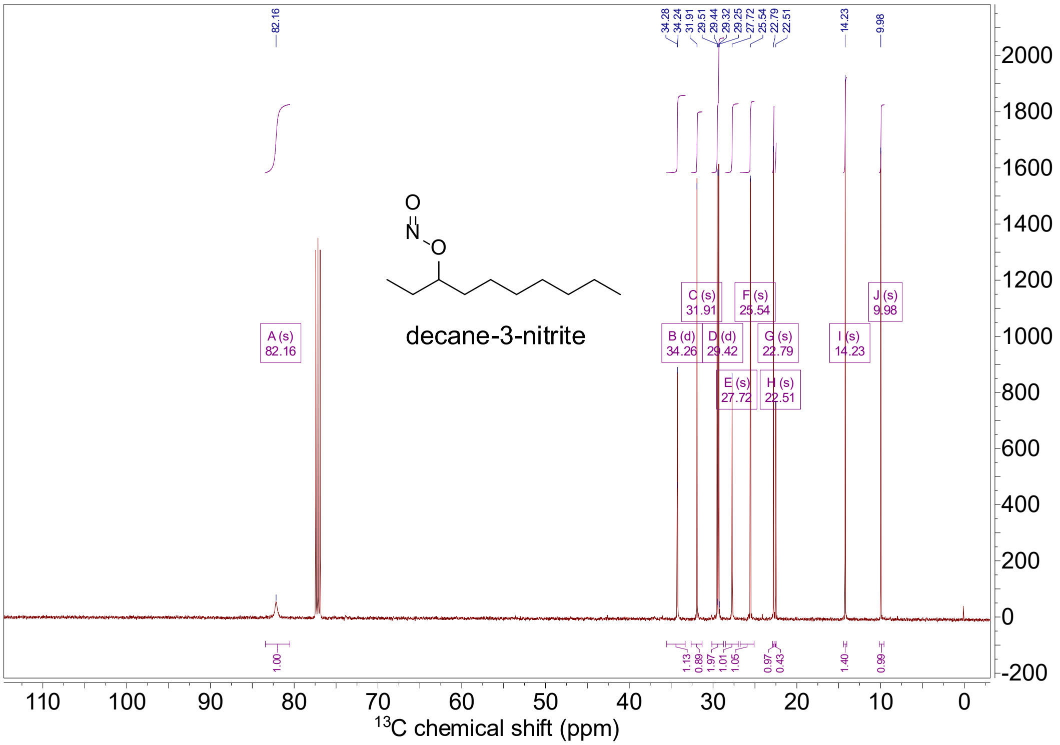

Figure S15. ^{13}C NMR spectrum of 3-decane nitrite.

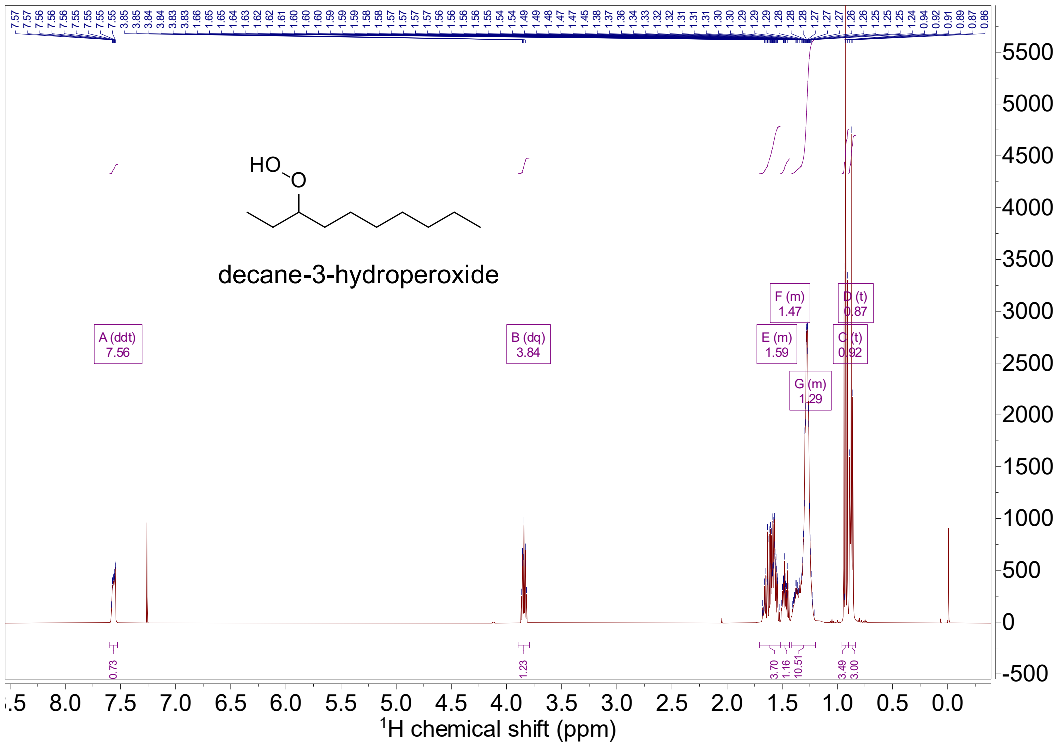

Figure S16. ^{1}H NMR spectrum of decane-3-hydroperoxide.

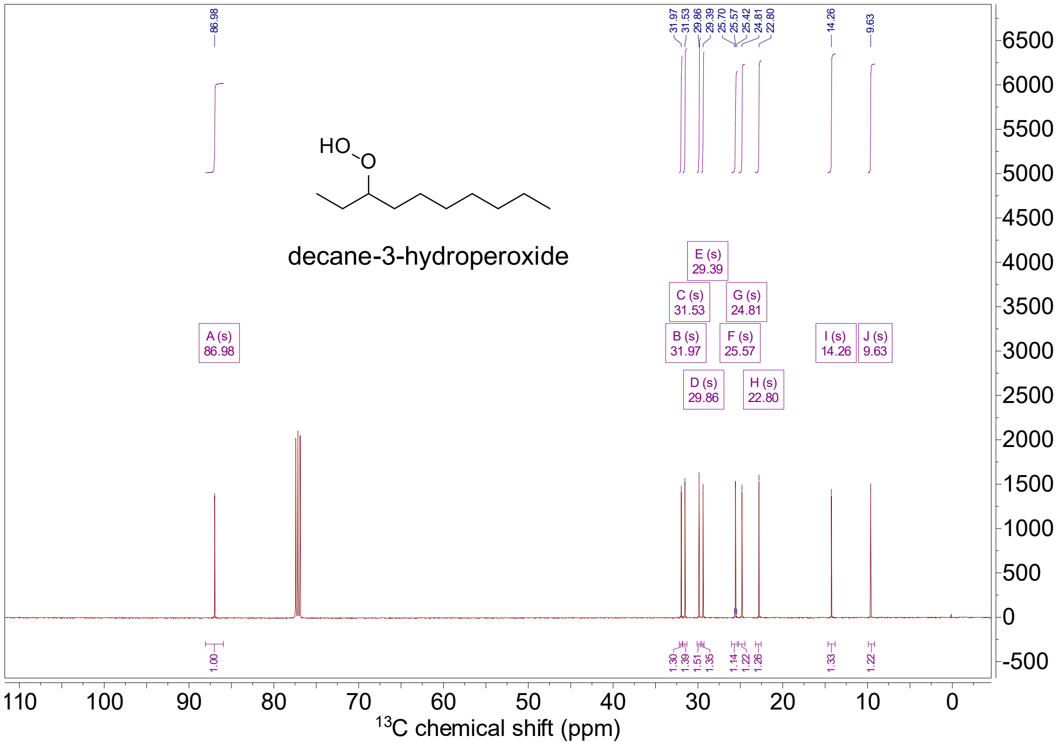

Figure S17. ^{13}C NMR spectrum of decane-3-hydroperoxide.

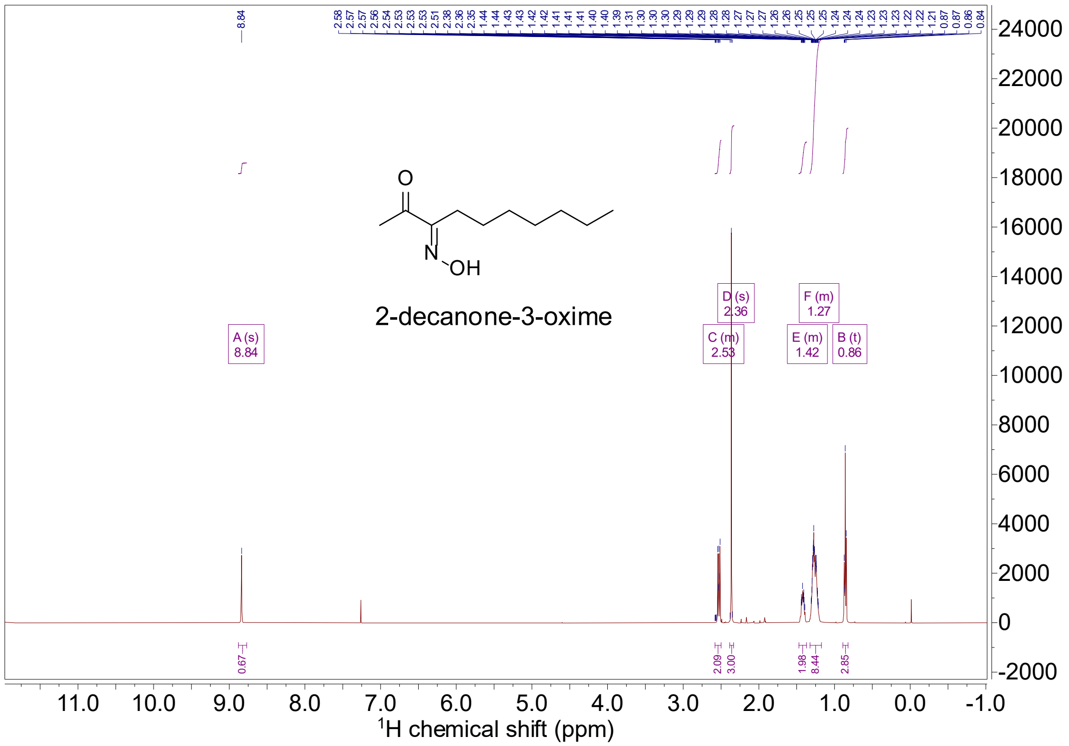

Figure S18. ^{1}H NMR spectrum of 2-decanone-3-oxime.

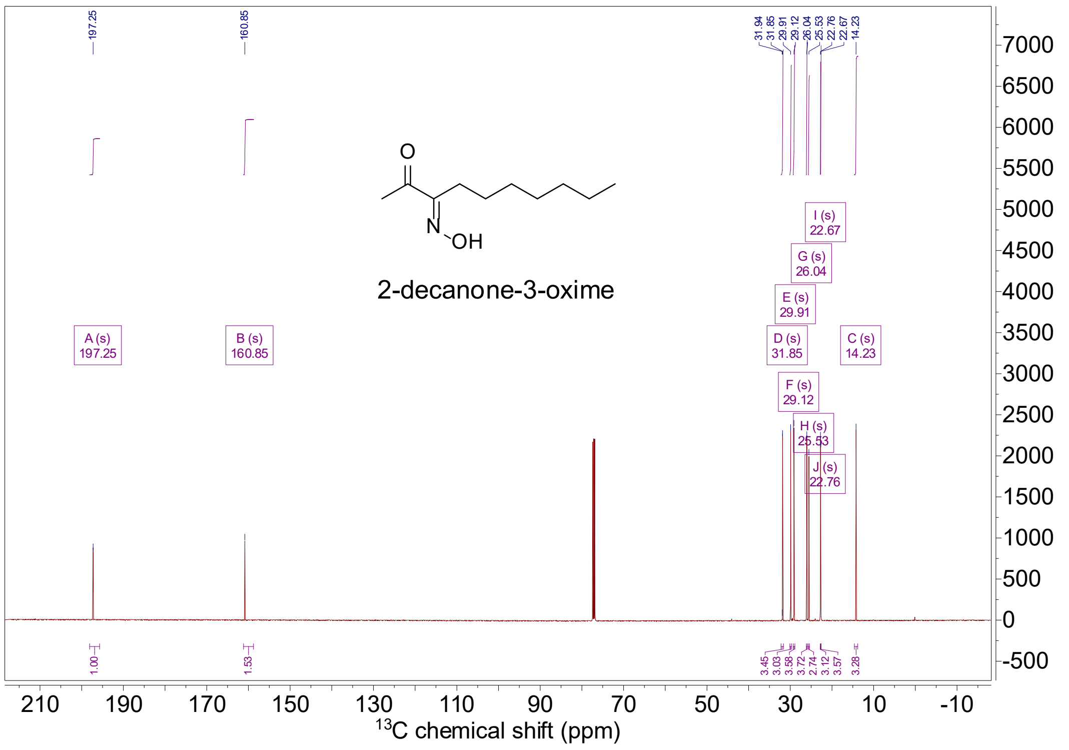

Figure S19. ^{13}C NMR spectrum of 2-decanone-3-oxime.

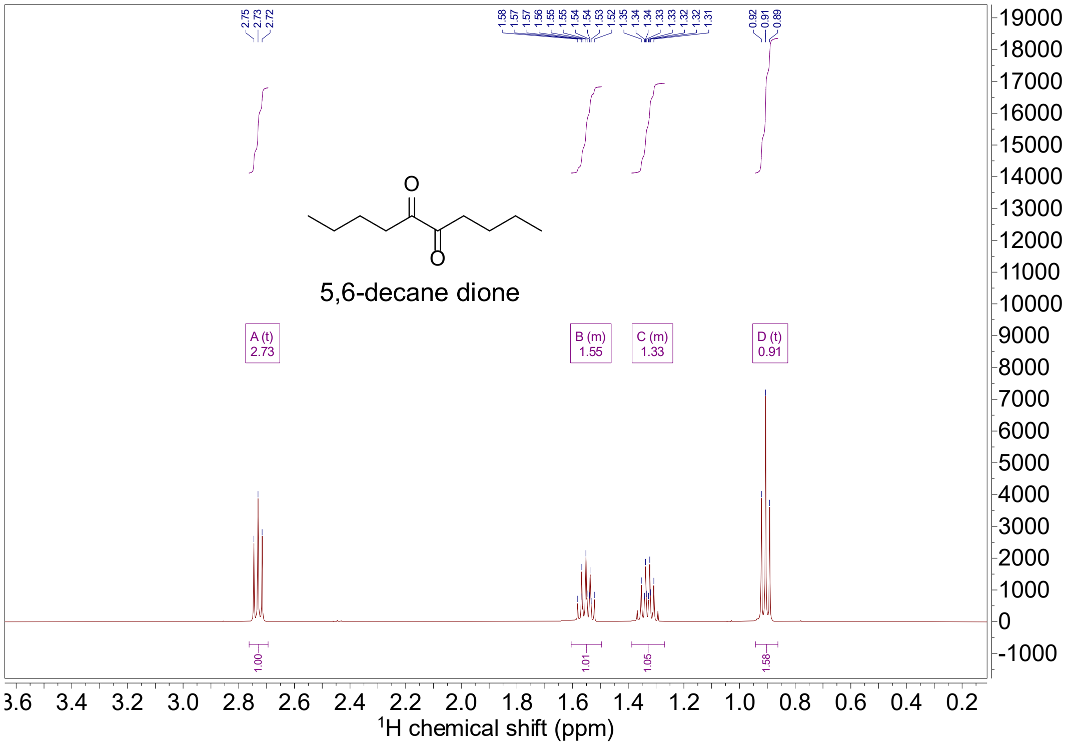

Figure S20. ^{1}H NMR spectrum of 5,6-decane dione.

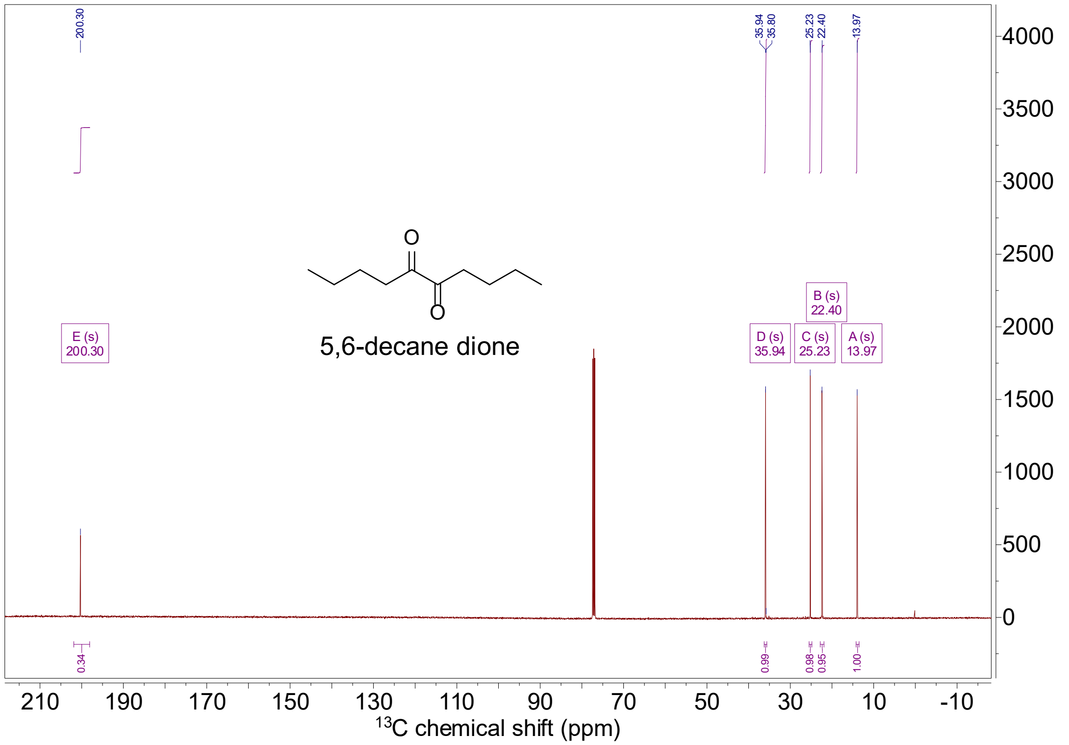

Figure S21. ^{13}C NMR spectrum of 5,6-decane dione.

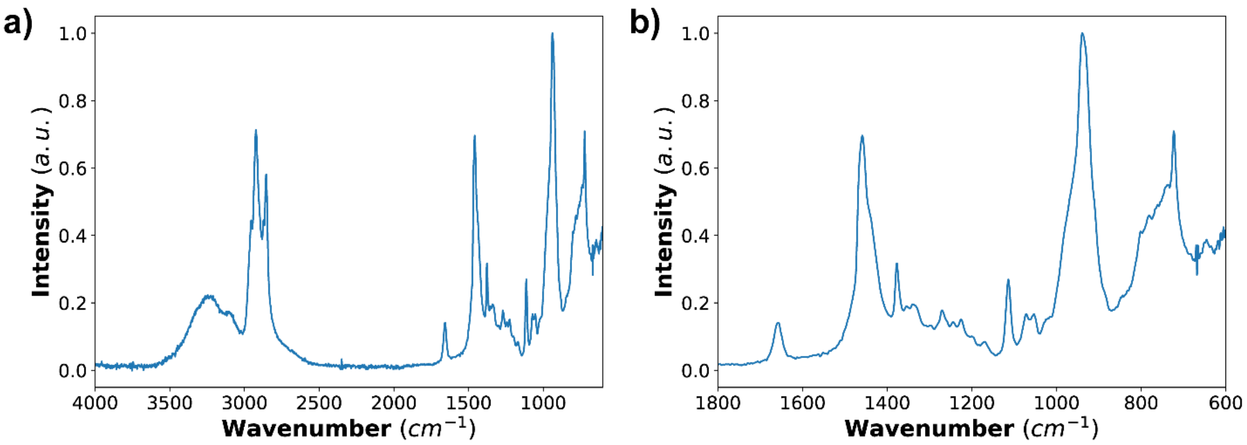

Figure S22. Infrared (IR) spectrum of 3-decanone oxime (a) entire range (b) zoom-in.

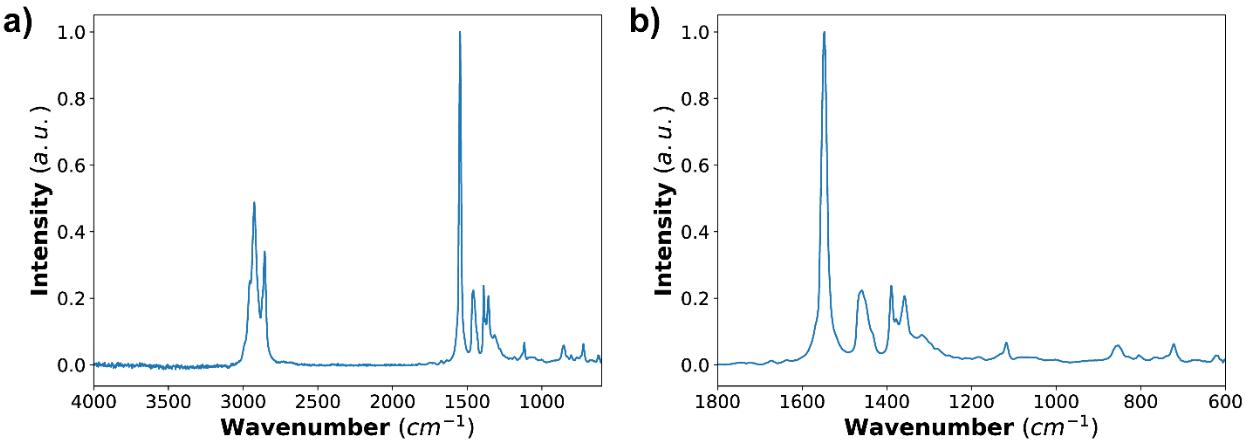

Figure S23. Infrared (IR) spectrum of 2-nitrodecane (a) entire range (b) zoom-in.

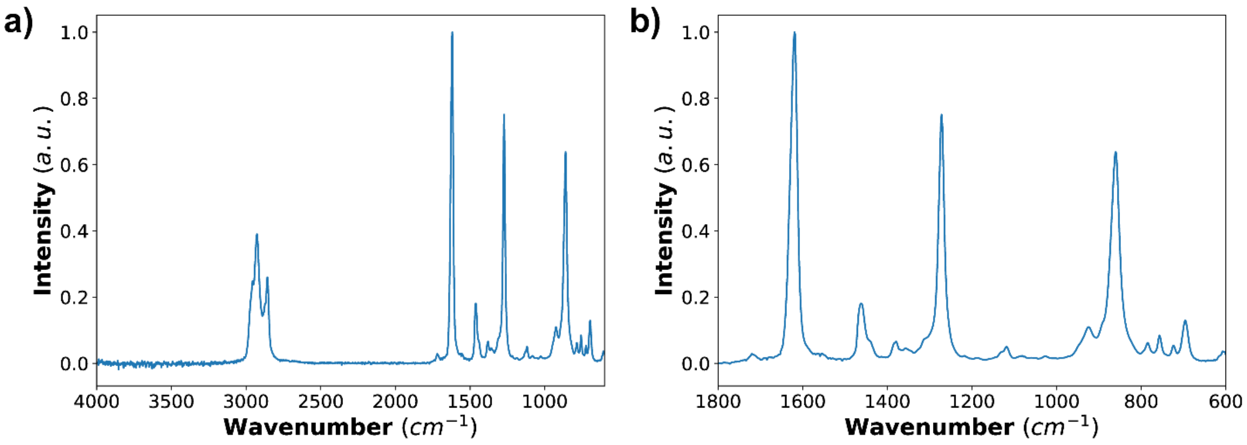

Figure S24. Infrared (IR) spectrum of decane-3-nitrate (a) entire range (b) zoom-in.

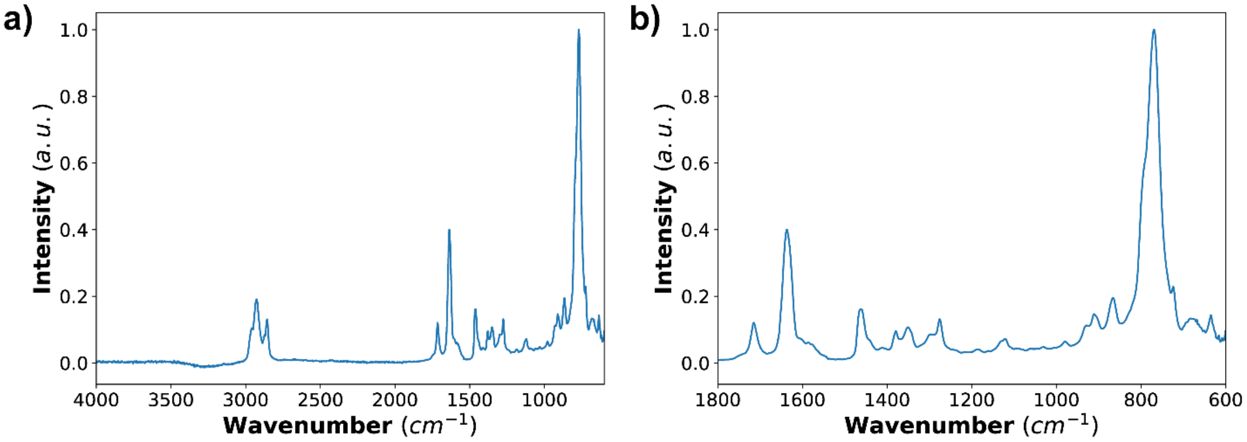

Figure S25. Infrared (IR) spectrum of decane-3-nitrite (a) entire range (b) zoom-in.

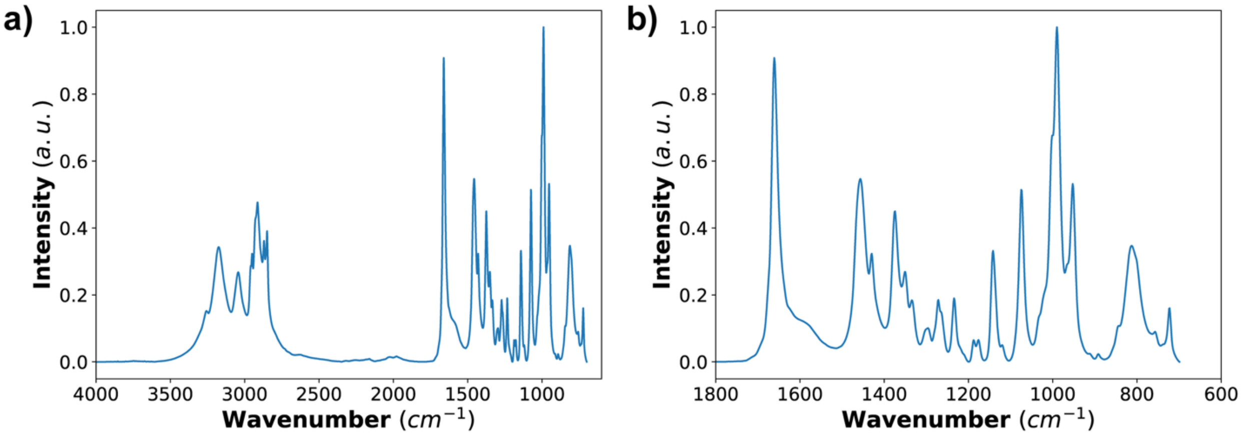

Figure S26. Infrared (IR) spectrum of 2-decanone-3-oxime. (a) entire range (b) zoom-in.

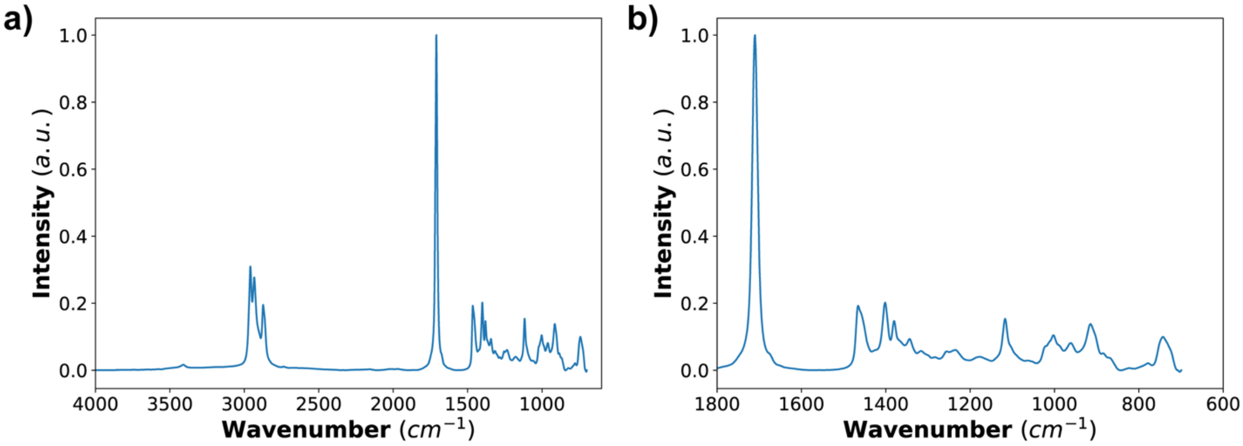

Figure S27. Infrared (IR) spectrum of 5,6-decanone (a) entire range (b) zoom-in.

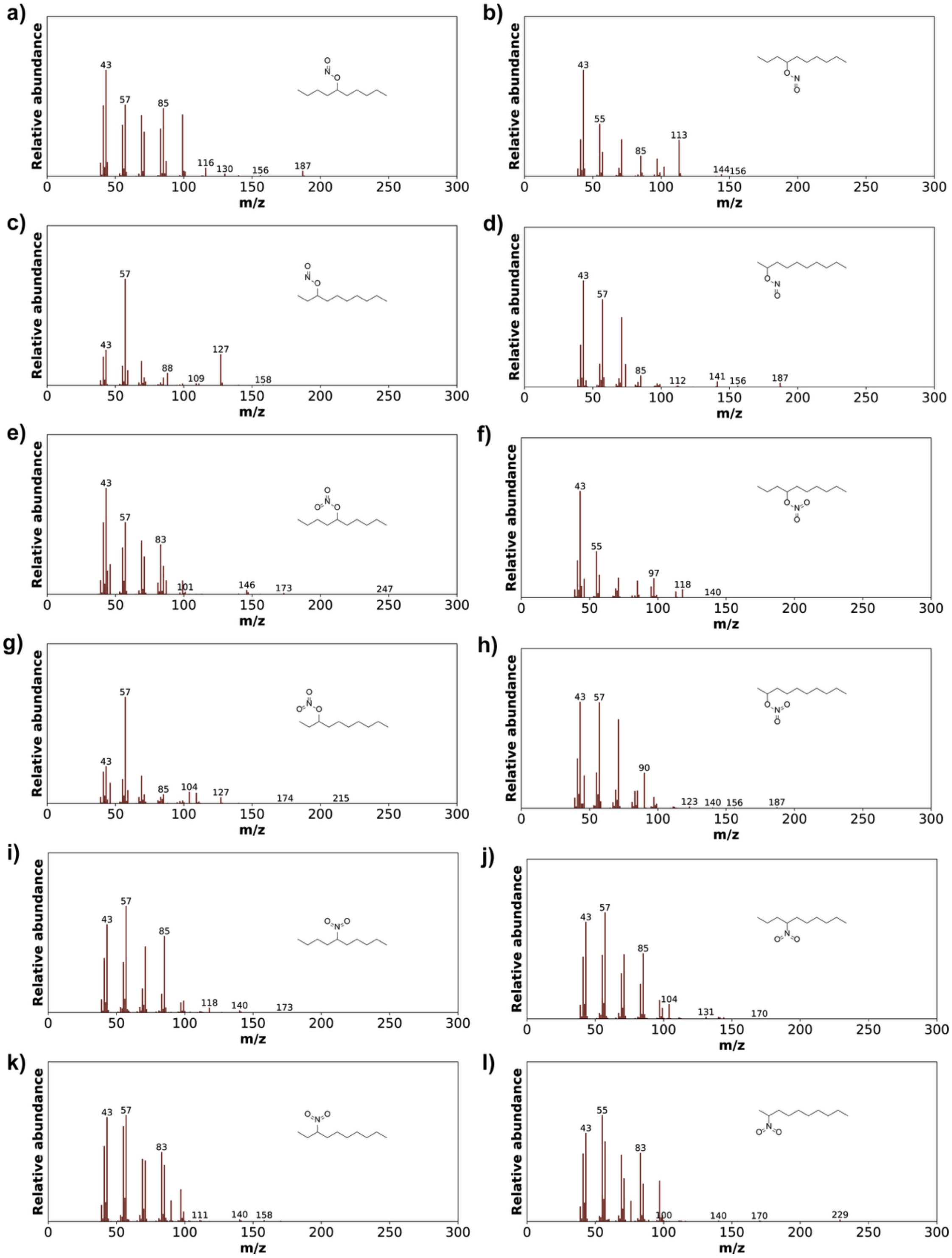

Figure S28. Mass spectra decane-5-nitrite (a), decane-4-nitrite (b), decane-3-nitrite (c), decane-2-nitrate (d), decane-5-nitrate (e), decane-4-nitrate (f), decane-3-nitrate (g), decane-2-nitrate (h), 5-nitrodecane (i), 4-nitrodecane (j), 3-nitrodecane (k), 2-nitrodecane (m).

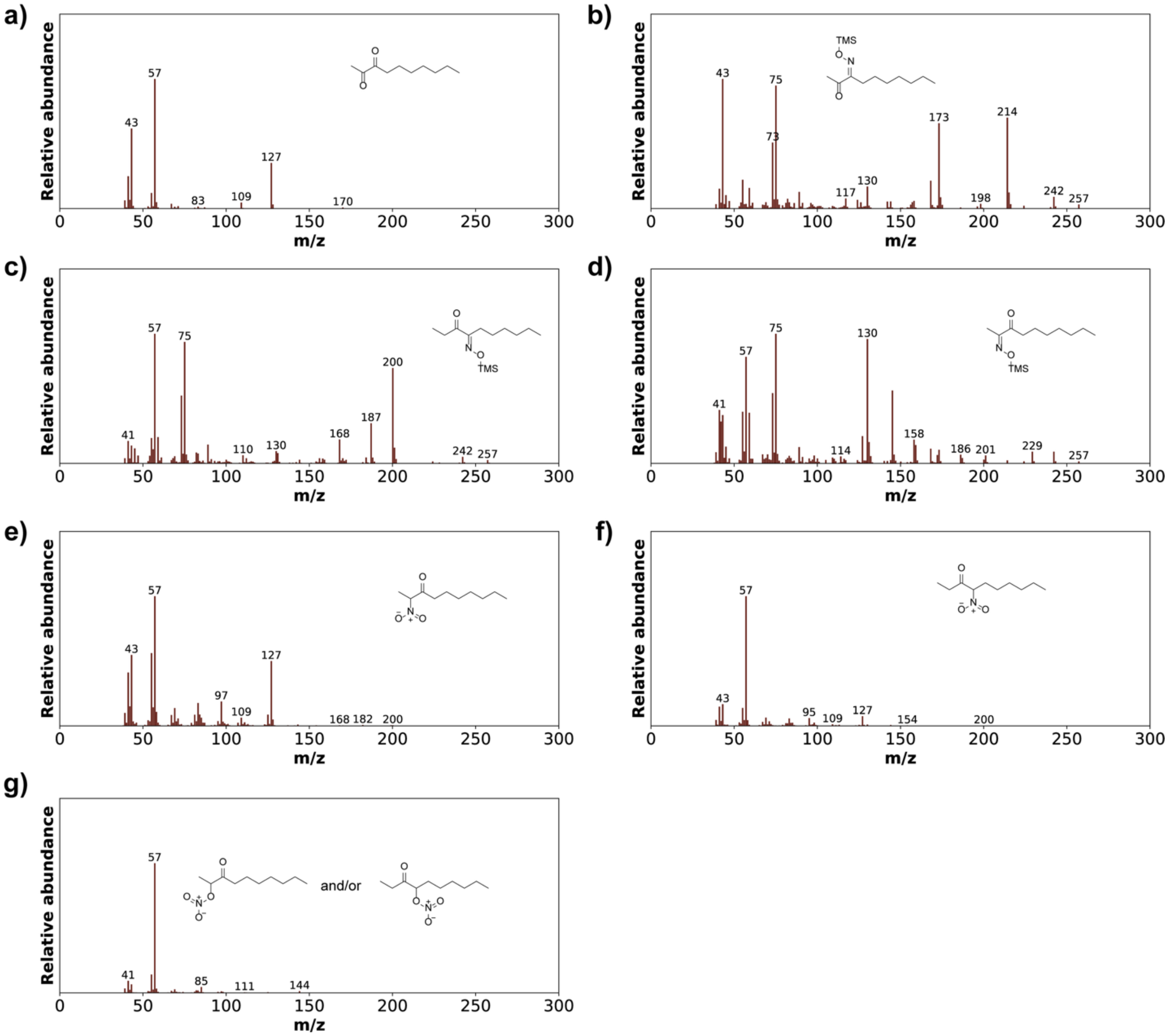

Figure S29. Mass spectra of 2,3-decane dione (a), decane-2-one-3 oxime trimethyl silyl (TMS) (b), decane-3-one-4-oxime TMS (c) and decane-3-one-2-oxime TMS (d) 2-nitro-3-decanone (e) 4-nitro-3-decanone (f) and α-nitrate-3-decanone.

6. DENSITY FUNCTIONAL THEORY (DFT) CALCULATIONS

All DFT calculations were performed using the M06-2X functional[15] and the aug-cc-pVTZ basis set[16,17] in the Gaussian 16 software.[18] Empirical dispersion effects were included in the form of Grimme's D3 correction without any damping.[19] All calculations were performed with an implicit n-decane solvent using the Self Consistent Reaction Field approach provided in Gaussian 16. All vibrational frequencies were scaled by a factor of 0.971, as recommended for this level of theory by Alecu et al.[20] All entropies were adjusted for a concentration of 1 mol/L. Contributions to the calculated enthalpies and entropies from vibrational modes < 100 cm^{-1} were adjusted using the rigid-rotor harmonic oscillator treatments of Head-Gordon[21] and Grimme,[22] respectively, as implemented in the GoodVibes software.[23] To account for solvation effects on the entropies of all species, the THERMO software,[24] which employs the method laid out by Garza ,[25] was used to estimate corrections for the rotational, translational, and solvent cavitation entropies in n-decane. The value for the cavitation entropy derived by incorporating both the relative permittivity and the isobaric thermal expansion coefficient was selected from THERMO.

All enthalpy (H), entropy (S), and Gibbs free energy (G) values are reported for each structure, with numbering corresponding to the Cartesian coordinates file. Values were calculated at 140 °C and a standard-state concentration of 1 mol L^{-1} using GoodVibes and are reported in Hartree units. The "Imag. Freq." column lists the single imaginary frequency associated with transition-state structures. The final two columns report the solvent entropy correction (from THERMO) and the resulting corrected Gibbs free energies.

	GoodVibes (Hartree)				THERMO (Hartree)	
**Structure**	**Enthalpy**	**Entropy**	**Free Energy**	**Imag. Freq. (cm^-1)**	**Entropy Correction**	**Corrected Free Energy**
1	-236.83654	0.06161	-236.89816		-0.00717	-236.89099
2	-236.18579	0.06367	-236.24946		-0.00721	-236.24225
3	-441.35409	0.07154	-441.42563		-0.00735	-441.41828
4	-441.34667	0.07312	-441.41978		-0.00740	-441.41238
5	-386.56175	0.06987	-386.63162		-0.00727	-386.62434
6	-516.47773	0.07736	-516.55509		-0.00761	-516.54748
7	-591.65925	0.07918	-591.73843		-0.00759	-591.73084
8	-387.19038	0.07053	-387.26091		-0.00729	-387.25362
9	-311.39902	0.06602	-311.46505		-0.00721	-311.45783
10	-515.83121	0.07904	-515.91024		-0.00757	-515.90267
11	-591.01175	0.08106	-591.09281		-0.00761	-591.08521
12	-386.54312	0.07265	-386.61578		-0.00729	-386.60849
13	-516.52834	0.07490	-516.60324		-0.00748	-516.59576
14	-310.88434	0.06504	-310.94938		-0.00724	-310.94215
15	-310.86486	0.06472	-310.92958		-0.00712	-310.92247
16	-310.24451	0.06518	-310.30968		-0.00721	-310.30247
17	-461.14290	0.07426	-461.21716		-0.00716	-461.21000
18	-515.93422	0.07687	-516.01108		-0.00743	-516.00365
19	-515.39082	0.07426	-515.46509		-0.00742	-515.45766

20	-515.92733	0.07831	-516.00565		-0.00743	-515.99821
21	-460.60083	0.07229	-460.67312		-0.00732	-460.66580
22	-515.38694	0.07527	-515.46221		-0.00746	-515.45475
23	-590.51732	0.08031	-590.59762		-0.00765	-590.58998
24	-665.69881	0.08197	-665.78078		-0.00759	-665.77319
25	-515.37163	0.07688	-515.44852		-0.00741	-515.44110
26	-590.50439	0.08046	-590.58485		-0.00757	-590.57728
27	-665.68504	0.08241	-665.76746		-0.00755	-665.75990
28	-385.44362	0.06874	-385.51236		-0.00730	-385.50506
29	-514.75434	0.07735	-514.83169		-0.00743	-514.82426
30	-589.88793	0.08072	-589.96865		-0.00767	-589.96098
31	-665.06756	0.08262	-665.15019		-0.00764	-665.14254
32	-590.56956	0.07695	-590.64651		-0.00754	-590.63897
33	-590.55535	0.07876	-590.63410		-0.00752	-590.62658
34	-589.93456	0.07867	-590.01323		-0.00761	-590.00562
35	-384.92104	0.06655	-384.98759		-0.00732	-384.98027
NO2	-205.06069	0.03610	-205.09679		-0.00548	-205.09131
O2	-150.31635	0.02920	-150.34554		-0.00528	-150.34026
NO	-129.88439	0.02922	-129.91361		-0.00526	-129.90835
NO3	-280.19490	0.03999	-280.23489		-0.00554	-280.22934
OH	-75.72272	0.02499	-75.74771		-0.00529	-75.74242
HNO2	-205.67149	0.03510	-205.70659		-0.00553	-205.70106
NO2H	-205.68458	0.03696	-205.72154		-0.00565	-205.71590
HO2	-150.89089	0.03328	-150.92417		-0.00545	-150.91872
HNO	-130.45580	0.03191	-130.48771		-0.00544	-130.48227
C5H12	-197.55871	0.05579	-197.61449		-0.00673	-197.60776
C5H11	-196.90797	0.05762	-196.96559		-0.00678	-196.95881
ts_1_2_no2h	-441.87973	0.08165	-441.94573	-1549.40	-0.00744	-441.93829
ts_1_2_hno2	-441.86779	0.08176	-441.94430	-1349.41	-0.00747	-441.93683
ts_1_2_ho2	-387.08805	0.07873	-387.16429	-1206.02	-0.00734	-387.15695
ts_1_2_hno	-366.64752	0.07984	-366.72427	-843.60	-0.00739	-366.71688
ts_5_8	-584.09581	0.10167	-584.19748	-1914.60	-0.00770	-584.18978
ts_4_14	-646.37348	0.08788	-646.46136	-1722.53	-0.00742	-646.45394
ts_8_12	-592.22012	0.08718	-592.30730	-1711.73	-0.00745	-592.29985
ts_7_11	-796.68716	0.09486	-796.78202	-1574.40	-0.00748	-796.77454
ts_6_10	-721.50670	0.09321	-721.59991	-1847.79	-0.00743	-721.59248
ts_14_16	-515.91272	0.08054	-515.99326	-1849.73	-0.00755	-515.98571
ts_15_16	-515.91488	0.08054	-515.99541	-6080.16	-0.00756	-515.98785
ts_15_17	-461.14088	0.07458	-461.21546	-514.93	-0.00733	-461.20813
ts_15_18	-515.92386	0.07770	-516.00156	-335.30	-0.00748	-515.99408
ts_15_20	-515.91814	0.07807	-515.99621	-282.93	-0.00747	-515.98874

ts_22_29	-720.41673	0.09121	-720.50794	-1925.51	-0.00749	-720.50045
ts_25_29	-720.42296	0.09229	-720.51525	-3759.89	-0.00758	-720.50767
ts_24_31	-870.72371	0.09719	-870.82090	-2082.86	-0.00748	-870.81342
ts_23_30	-795.54653	0.09558	-795.64211	-2042.02	-0.00750	-795.63461
ts_27_31	-870.73369	0.09708	-870.83077	-4196.14	-0.00757	-870.82320
ts_26_30	-795.55379	0.09580	-795.64959	-4112.70	-0.00761	-795.64198
ts_13_14	-721.55546	0.09052	-721.64598	-1877.93	-0.00741	-721.63857
ts_32_34	-795.59626	0.09288	-795.68914	-2095.69	-0.00742	-795.68172

7. RADICAL INHIBITION EXPERIMENT WITH BUTYLATED HYDROXY TOLUENE

To obtain more direct evidence for the involvement of carbon-centered radical intermediates, we considered both radical inhibition and radical trapping experiments. However, the reaction conditions used in this study feature large concentrations of open-shell radical species (O_2, NO, NO_2) that make conventional "radical trapping" experiments challenging. These species are themselves very efficient radical traps.

A radical inhibition experiment was conducted using butylated hydroxytoluene (BHT) as a radical scavenger to obtain more direct evidence for the involvement of carbon-centered radical intermediates (**Figure S30**). BHT was introduced after 40 min, and the formation of primary oxidation products was compared to a control reaction. Under these conditions, no significant change in product distribution was observed beyond experimental uncertainty. This inconclusive result is not interpreted as support for a non-radical mechanism. The NO flow rate (~0.2 mmol·min^{-1}) and the estimated radical content in both the gas phase (ca. 2.4 mmol in the headspace) and solution (unknown) suggest the 1 mmol of BHT added is insufficient to quench the radical population, which is rapidly replenished under the NO_x/O_2 conditions. Higher loadings of BHT were considered; however, the 1 mmol used is already 2 mol% relative to the decane solvent (and reactant). Higher BHT loadings will significantly change the reaction composition and didn't seem justified.

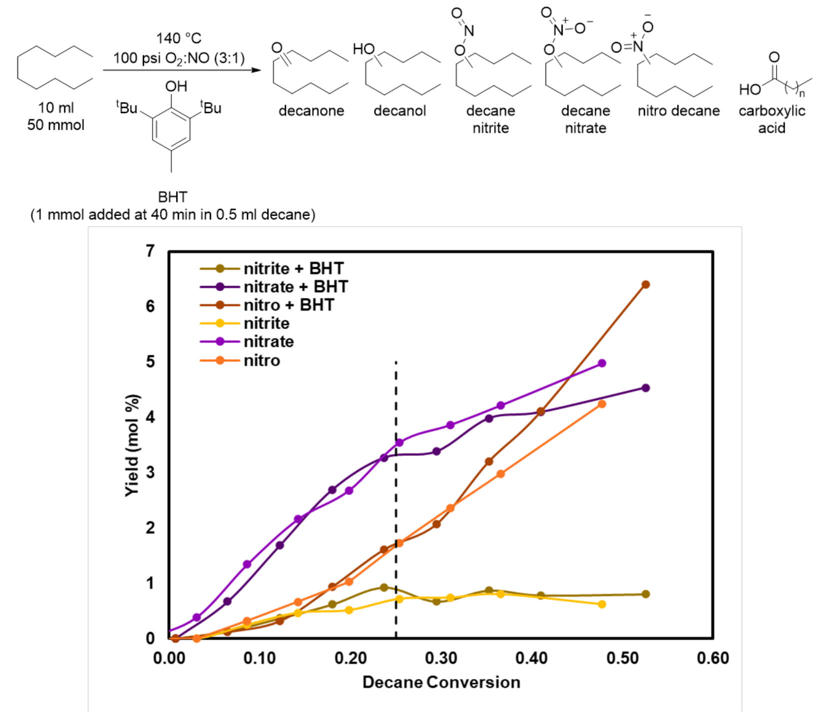

Figure S30. Radical inhibition experiment with butylated hydroxytoluene (decane, 140 °C 100 psi, NO/N_2 = flow (20% NO in N_2) = 25 mL/min, O_2 flow = 15 mL/min).

Conventional radical trapping experiments were also considered. However, the high concentrations of O_2 and NO_2 in our reactions are very efficient (diffusion-controlled) radical traps that will compete with typical trapping reagents. Moreover, NO_2 is a reactive radical (more so than O_2) that will react directly with many trapping agents. Notably, the nitrate-, nitrite-, and nitro-containing products observed in this work (–ONO, –NO_2, –ONO_2) are well-established products of radical trapping by nitrogen oxides and provide evidence

for the presence of carbon-centered radical intermediates. Additional discussion of these processes can be found in the literature.[26,27]

8. GPC MEASUREMENTS

Efforts were made to monitor changes in the molecular weight of HDPE during oxidation using gel permeation chromatography (GPC). The measurements were performed in 1,2,4-trichlorobenzene at 160 °C on an instrument designed for polyolefin substrates. We found that the molecular weight decreases rapidly and falls below the lower molecular weight detection limit of our instrument after only a single time point, preventing meaningful time-resolved analysis (see representative traces below, where the first 30 min sample shows a significant decrease and broadening of the molecular weight relative to the starting PE sample). Additionally, we noted that during sample preparation and analysis, the colorless polymers samples had turned dark brown and precipitates were observed. We tentatively attribute this behavior to the presence of nitrogen-containing functionalities introduced during oxidation, which may exhibit limited thermal stability under the GPC operating conditions. Given these observations, the molecular weight values obtained from GPC should be interpreted with caution.

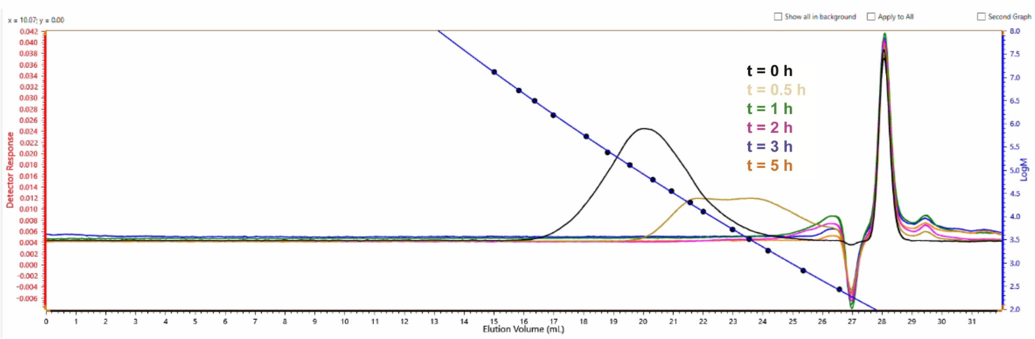

Figure S31. GPC measurements of the polyethylene oxidation experiments as function of time (120 °C, P_{O2} = 8 bar, P_{NO} = 4 bar, P_{N2} = 28 bar).

9. REFERENCES

(1) Scanlon, J. T.; Willis, D. E. Calculation of Flame Ionization Detector Relative Response Factors Using the Effective Carbon Number Concept. *J. Chromatogr. Sci.* **1985**, *23* (8), 333–340. https://doi.org/10.1093/chromsci/23.8.333.

(2) Jorgensen, A. D.; Picel, K. C.; Stamoudis, V. C. Prediction of Gas Chromatography Flame Ionization Detector Response Factors from Molecular Structures. *Anal. Chem.* **1990**, *62* (7), 683–689. https://doi.org/10.1021/ac00206a007.

(3) Poole, C. F.; Poole, S. K. Instrumental Aspects of Gas Chromatography. In *The Essence of Chromatography*; 1991; pp 231–309. https://doi.org/10.1016/b978-0-444-88492-3.50006-1.

(4) Otten, M.; Klein Gebbink, I.; Schara, P. J.; Tomovic´, Z.; Lutz, M.; Bruijnincx, P. C. A.; Thevenon, A. Post-Polymerization Modification of Polyethylene through Photochemical Oximation and Consecutive Ketonization. *J. Am. Chem. Soc.* **2025**, *147*, 22827–22838. https://doi.org/10.1021/jacs.5c05212.

(5) Tanemura, K. Silica Gel-Mediated Hydrohalogenation of Unactivated Alkenes Using Hydrohalogenic Acids under Organic Solvent-Free Conditions. *Tetrahedron Lett.* **2018**, *59* (49), 4293–4298. https://doi.org/10.1016/j.tetlet.2018.10.043.

(6) Möller, M.; Hentschel, C.; Chi, L.; Studer, A. Aggregation Behaviour of Peptide-Polymer Conjugates Containing Linear Peptide Backbones and Multiple Polymer Side Chains Prepared by Nitroxide-Mediated Radical Polymerization. *Org. Biomol. Chem.* **2011**, *9* (7), 2403. https://doi.org/https://doi.org/10.1039/C0OB01047B.

(7) Schneider, M.; Ballschmiter, K. Separation of Diastereomeric and Enantiomeric Alkyl Nitrates - Systematic Approach to Chiral Discrimination on Cyclodextrin LIPODEX-D. *Chem. - A Eur. J.* **1996**, *2* (5), 539–544. https://doi.org/10.1002/chem.19960020513.

(8) Suzuki, H.; Takeuchi, T.; Mori, T. Direct Oxidation of Methyl Ethers to Carbonyl Compounds with a Combination of Nitrogen Dioxide and Water in the Presence or Absence of Ozone. *Bull. Chem. Soc. Jpn.* **1997**, *70* (12), 3111–3115. https://doi.org/10.1246/bcsj.70.3111.

(9) Grossi, L.; Strazzari, S. A New Synthesis of Alkyl Nitrites: The Reaction of Alkyl Alcohols with Nitric Oxide in Organic Solvents. *J. Org. Chem.* **1999**, *64* (22), 8076–8079. https://doi.org/10.1021/jo982341p.

(10) Rüedi, G.; Oberli, M. A.; Nagel, M.; Weymuth, C.; Hansen, H. J. A Practical and User-Friendly Method for the Selenium-Free One-Step Preparation of 1,2-Diketones and Their Monoxime Analogs. *Synlett* **2004**, *4* (13), 2315–2318. https://doi.org/10.1055/s-2004-832815.

(11) Guan, H.; Sun, S.; Mao, Y.; Chen, L.; Lu, R.; Huang, J.; Liu, L. Iron (II) -Catalyzed Site-Selective Functionalization of Unactivated C(Sp3)-H Bonds Guided by Alkoxy Radicals. *Angew. Chem.* **2018**, *130* (35), 11583–11587.

(12) Shiratori, Y.; Jiang, J.; Kubota, K.; Maeda, S.; Ito, H. Ring Expansion of Cyclic Boronates via Oxyboration of Arynes. *J. Am. Chem. Soc.* **2024**, *146* (3), 1765–1770. https://doi.org/10.1021/jacs.3c11851.

(13) Lukashev, M.; Pushechnikov, A.; Demin, P.; Denton, K. WO2022047014A1.Pdf. WO 2022/047014 A1, 2022.

(14) Parkinson, E. I.; Jason Hatfield, M.; Tsurkan, L.; Hyatt, J. L.; Edwards, C. C.; Hicks, L. D.; Yan, B.; Potter, P. M. Requirements for Mammalian Carboxylesterase Inhibition by Substituted Ethane-

1,2-Diones. *Bioorganic Med. Chem.* **2011**, *19* (15), 4635–4643. https://doi.org/10.1016/j.bmc.2011.06.012.

(15) Zhao, Y.; Truhlar, D. G. The M06 Suite of Density Functionals for Main Group Thermochemistry, Thermochemical Kinetics, Noncovalent Interactions, Excited States, and Transition Elements: Two New Functionals and Systematic Testing of Four M06-Class Functionals and 12 Other Function. *Theor. Chem. Acc.* **2008**, *120*, 215–241. https://doi.org/10.1007/s00214-007-0310-x.

(16) Dunning, T. H. Gaussian Basis Sets for Use in Correlated Molecular Calculations. I. The Atoms Boron through Neon and Hydrogen. *J. Chem. Phys.* **1989**, *90*, 1007–1023. https://doi.org/10.1063/1.456153.

(17) Kendall, R. A.; Dunning, T. H.; Harrison, R. J. Electron Affinities of the First-row Atoms Revisited. Systematic Basis Sets and Wave Functions. *J. Chem. Phys.* **1992**, *96*, 6796–6806.

(18) Frisch, M. J.; Trucks, G. W.; Schlegel, H. B.; Scuseria, G. E.; Robb, M. A., Cheeseman, J. R.; Scalmani, G.; Barone, V.; Petersson, G. A.; Nakatsuji, H.; Li, X.; Caricato, M.; Marenich, A. V.; Bloino, J.; Janesko, B. G.; Gomperts, R.; Mennucci, B.; Hratchian, H. P.; Ortiz, J. V.; Izmaylov, A. F.; Sonnenberg, J. L.; Williams-Young, D.; Ding, F.; Lipparini, F.; Egidi, F.; Goings, J.; Peng, B.; Petrone, A.; Henderson, T.; Ranasinghe, D.; Zakrzewski, V. G.; Gao, J.; Rega, N.; Zheng, G.; Liang, W.; Hada, M.; Ehara, M.; Toyota, K.; Fukuda, R.; Hasegawa, J.; Ishida, M.; Nakajima, T.; Honda, Y.; Kitao, O.; Nakai, H.; Vreven, T.; Throssell, K.; Montgomery, J. A., J.; Peralta, J. E.; Ogliaro, F.; Bearpark, M. J.; Heyd, J. J.; Brothers, E. N.; Kudin, K. N.; Staroverov, V. N.; Keith, T. A.; Kobayashi, R.; Normand, J., Raghavachari, K.; Rendell, A. P.; Burant, J. C.; Iyengar, S. S.; Tomasi, J.; Cossi, M.; Millam, J. M.; Klene, M.; Adamo, C.; Cammi, R.; Ochterski, J. W.; Martin, R. L.; Morokuma, K.; Farkas, O.; Foresman, J. B.; Fox, D. J. Gaussian 16, Revision C.01. Gaussian, Inc.: Wallingford CT 2019.

(19) Grimme, S.; Antony, J.; Ehrlich, S.; Krieg, H. A Consistent and Accurate Ab Initio Parametrization of Density Functional Dispersion Correction (DFT-D) for the 94 Elements H-Pu. *J. Chem. Phys.* **2010**, *132*, 154104. https://doi.org/10.1063/1.3382344.

(20) Alecu, I. M.; Zheng, J.; Zhao, Y.; Truhlar, D. G. Computational Thermochemistry: Scale Factor Databases and Scale Factors for Vibrational Frequencies Obtained from Electronic Model Chemistries. *J. Chem. Theory Comput.* **2010**, *6*, 2872–2887. https://doi.org/10.1021/ct100326h.

(21) Li, Y.-P.; Gomes, J.; Sharada, S. M.; Bell, A. T.; Head-Gordon, M. Improved Force-Field Parameters for QM/MM Simulations of the Energies of Adsorption for Molecules in Zeolites and a Free Rotor Correction to the Rigid Rotor Harmonic Oscillator Model for Adsorption Enthalpies. *J. Phys. Chem. C.* **2015**, *119*, 1840–1850. https://doi.org/10.1021/jp509921r.

(22) Grimme, S. Supramolecular Binding Thermodynamics by Dispersion-Corrected Density Functional Theory. *Chem. Eur. J.* **2012**, *18*, 9955–9964. https://doi.org/10.1002/chem.201200497.

(23) Luchini, G.; Alegre-Requena, J. V.; Funes-Ardoiz, I.; Paton, R. S. GoodVibes: Automated Thermochemistry for Heterogeneous Computational Chemistry Data. *F1000Research* **2020**, *9*, 291. https://doi.org/10.12688/f1000research.22758.1.

(24) Conti, S.; Cecchini, M. Predicting Molecular Self-Assembly at Surfaces: A Statistical Thermodynamics and Modeling Approach. *Phys. Chem. Chem. Phys.* **2016**, *18*, 31480–31493. https://doi.org/10.1039/C6CP05249E.

(25) Garza, A. J. Solvation Entropy Made Simple. *J. Chem. Theory Comput.* **2019**, *15*, 3204–3214. https://doi.org/10.1021/acs.jctc.9b00214.

(26) Hartung, J. Organic Radical Reactions Associated with Nitrogen Monoxide. *Chem. Rev.* **2009**, *109* (9), 4500–4517. https://doi.org/10.1021/cr900085j.

(27) Gray, P.; Yoffe, A. D. The Reactivity And Structure Of Nitrogen Dioxide. *Chem. Rev.* **1955**, *55* (6), 1069–1154.
